# Seed- and leaf-based expression of FGF21-transferrin fusion proteins for oral delivery and treatment of non-alcoholic steatohepatitis

**DOI:** 10.3389/fpls.2022.998596

**Published:** 2022-09-29

**Authors:** Hsuan-Wu Hou, Christopher A. Bishop, Jana Huckauf, Inge Broer, Susanne Klaus, Henrik Nausch, Johannes F. Buyel

**Affiliations:** ^1^Department Bioprocess Engineering, Fraunhofer Institute for Molecular Biology and Applied Ecology IME, Aachen, Germany; ^2^Chair for Agrobiotechnology, University of Rostock, Rostock, Germany; ^3^Department of Physiology of Energy Metabolism, German Institute of Human Nutrition Potsdam-Rehbrücke, Nuthetal, Germany; ^4^Institute of Nutritional Science, University of Potsdam, Nuthetal, Germany; ^5^Institute of Molecular Biotechnology, RWTH Aachen University, Aachen, Germany; ^6^Department of Biotechnology (DBT), Institute of Bioprocess Science and Engineering (IBSE), University of Natural Resources and Life Sciences (BOKU), Vienna, Austria

**Keywords:** bioencapsulation, furin cleavage site, liver-targeting PLUS peptide, transferrin-mediated oral delivery, transient and stable transformation

## Abstract

Non-alcoholic steatohepatitis (NASH) is a global disease with no effective medication. The fibroblast growth factor 21 (FGF21) can reverse this liver dysfunction, but requires targeted delivery to the liver, which can be achieved *via* oral administration. Therefore, we fused FGF21 to transferrin (Tf) *via* a furin cleavage site (F), to promote uptake from the intestine into the portal vein, yielding FGF21-F-Tf, and established its production in both seeds and leaves of commercial *Nicotiana tabacum* cultivars, compared their expression profile and tested the bioavailability and bioactivity in feeding studies. Since biopharmaceuticals need to be produced in a contained environment, e.g., greenhouses in case of plants, the seed production was increased in this setting from 239 to 380 g m^–2^ a^–1^ seed mass with costs of 1.64 € g^–1^ by side branch induction, whereas leaves yielded 8,193 g m^–2^ a^–1^ leave mass at 0.19 € g^–1^. FGF21-F-Tf expression in transgenic seeds and leaves yielded 6.7 and 5.6 mg kg^–1^ intact fusion protein, but also 4.5 and 2.3 mg kg^–1^ additional Tf degradation products. Removing the furin site and introducing the liver-targeting peptide PLUS doubled accumulation of intact FGF21-transferrin fusion protein when transiently expressed in *Nicotiana benthamiana* from 0.8 to 1.6 mg kg^–1^, whereas truncation of transferrin (nTf338) and reversing the order of FGF21 and nTf338 increased the accumulation to 2.1 mg kg^–1^ and decreased the degradation products to 7% for nTf338-FGF21-PLUS. Application of partially purified nTf338-FGF21-PLUS to FGF21^–/–^ mice by oral gavage proved its transfer from the intestine into the blood circulation and acutely affected hepatic mRNA expression. Hence, the medication of NASH *via* oral delivery of nTf338-FGF21-PLUS containing plants seems possible.

## Introduction

To date, non-alcoholic steatohepatitis (NASH) is a widespread disease in developed countries with a total prevalence of 0.4 billion patients (Weiß et al., 2014; Estes et al., 2018; Asrani et al., 2019; Povsic et al., 2019; Younossi et al., 2019; Kasper et al., 2020). NASH is characterized by an excessive accumulation of fat in the liver, which induces inflammation, leading to cirrhosis and liver failure (LaBrecque et al., 2014). Besides liver transplantation, however, there are currently no approved NASH therapeutics available (Moustafa et al., 2016; Mullard, 2020; Albhaisi and Sanyal, 2021), and medication is limited to symptom mitigation (Sharma et al., 2021).

A recently proposed drug candidate for the treatment of NASH is the fibroblast growth factor 21 (FGF21) (Xu et al., 2009; Dushay et al., 2010; Verzijl et al., 2020), an autocrine hormone that induces fat oxidation and inhibits fat synthesis in the liver (Nishimura et al., 2000; Badman et al., 2007; Lundåsen et al., 2007; Feingold et al., 2012; Woo et al., 2013). Though, a limiting factor is the 1−2 h half-life of FGF21 in the blood stream (Kharitonenkov et al., 2005; Gimeno and Moller, 2014; Zhen et al., 2016; Sonoda et al., 2017), and FGF21 analogs with half-lives of 24 and 98 h were developed by fusing the protein to either IgG (PF-05231023, Pfizer) (Huang et al., 2013; Giragossian et al., 2015; Talukdar et al., 2016) or polyethylene glycol (BMS-986036, Bristol-Myers Squibb) (Sanyal et al., 2018; Charles et al., 2019; Verzijl et al., 2020). These analogs decreased the blood triglycerides up to 20% in clinical trials after subcutaneous or intravenous administration (Talukdar et al., 2016; Charles et al., 2019), but in the case of PF-05231023, led to off-target effects such as altered bone turnover (Talukdar et al., 2016). These off-target effects might be reduced if FGF21 is directed exclusively to the liver *via* oral delivery, rather than administered systemically, as proteins are absorbed in the intestine and directly transported to the liver *via* the portal vein before entering the blood circulation (Schwegler and Lucius, 2022).

In contrast to other recombinant production platforms such as bacteria, yeast, insect or mammalian cell cultures, transgenic plants can be used for oral administration without the need to purify the recombinant protein, since human pathogens do not replicate in plants (Ghag et al., 2021). For example, feeding tobacco leaves, expressing exendin-4 (Ex-4), to diabetic mice led to a 25% reduction in blood glucose levels (Kwon et al., 2013), whereby the uptake of Ex-4 from the intestine into the blood stream was facilitated through a fusion with cholera toxin B (CTB), mediating the mucosal transfer *via* the ganglioside GM1 receptor. However, CTB is a strong immune trigger, which limits the possibility of long-term application. Therefore, Ex-4 was fused to the non-immunogenic human iron-carrier protein transferrin (Tf), enabling the uptake *via* the enterocyte Tf-receptor. When applied orally, Ex-4-Tf reduced blood glucose levels by 31% compared to the 25 and 20% achieved with Ex-4-CTB and unmodified Ex-4, respectively (Choi et al., 2014). In contrast, when the purified recombinant proteins were administered intraperitoneally, Ex-4-Tf was 12% less effective (44% reduction) than Ex-4 (56% reduction), showing that the Tf domain can affect the bioactivity of the fusion protein.

In this context, plant seeds have been recognized as particularly suitable for oral delivery in the past as they contain 80−350 g kg^–1^ protein per seed dry mass (SDM), compared to 10−20 g kg^–1^ protein per leaf fresh mass (LFM) [corresponding to 83−167 g kg^–1^ leaf dry mass (LDM)] (Buyel, 2016), and display a low protease activity in the development stage, resulting in high recombinant protein yields (Benchabane et al., 2008; Boothe et al., 2010). For example, whereas Tf accumulated to 0.14 g kg^–1^ LFM in tobacco leaves (Brandsma et al., 2010; Choi et al., 2014; Wang et al., 2014) [corresponding to 1.17 g kg^–1^ LDM], expression in rice seeds yielded 10.00 g kg^–1^ SDM (Zhang et al., 2010). Additionally, seeds produce few secondary metabolites that could be regarded as potential impurities and can be stored at ambient conditions after harvest for at least six years (Benchabane et al., 2008; Boothe et al., 2010; Cunha et al., 2011). Accordingly, commercial seed production in greenhouses has already been established for rice (Ventria Bioscience, Junction City, KS, USA^1^; Broz et al., 2013) and barley (ORF Genetics, Kópavogur, Island^2^), and seeds have been used for several oral vaccination trails. In some of the corresponding feeding studies, tobacco seeds were used because the production of recombinant proteins is well established in this system, and they are edible due to the absence of alkaloids such as nicotine (Rossi et al., 2013, 2014).

Here, we selected tobacco (*Nicotiana tabacum*) seeds of commercial cultivars Virginia Golta (VG) and SL632 (SL632) as a production platform as these were bred to high leaf and seed biomass (NiCoTa GmbH Rheinstetten, Germany^3^; Sunchem NL, Amsterdam, Netherlands^4^). We optimized the overall leaf and seed yield per unit area and time in a greenhouse and compared the biomass output of both production systems. For oral delivery, we fused FGF21 to Tf *via* a furin cleavage site and generated stably transformed plants, expressing the fusion protein with the constitutively active 35S promoter/terminator expression cassette from the Cauliflower Mosaic Virus (CaMV) (Odell et al., 1985; Kay et al., 1987). In parallel, we optimized the stability of the fusion protein by domain exchange and shuffling *via* transient expression in *Nicotiana benthamiana* using the same expression cassette. Finally, we analyzed the *in vivo* bioavailability and bioactivity of the optimized FGF21-Tf fusion protein by oral administration to FGF21 knockout (FGF21^–/–^) mice.

## Materials and methods

### Cloning of FGF21-transferrin expression constructs

**FIGURE 1:**
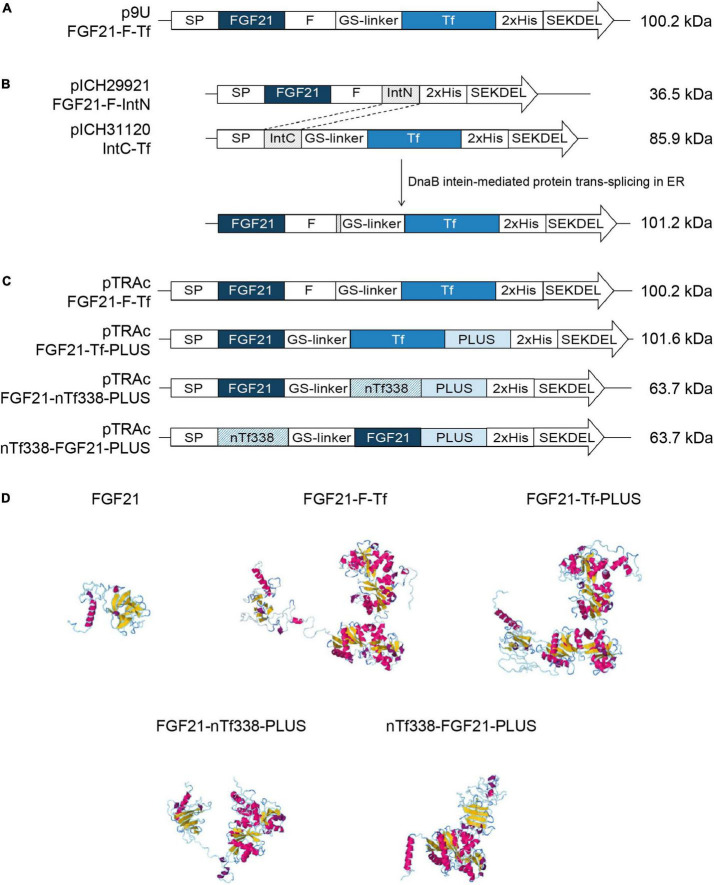
Constructs and models used for the stable and transient expression of FGF21-transferrin fusion proteins. **(A)** p9U stable expression vector containing FGF21-F-Tf in *N. tabacum* SL632 and VG. **(B)** MagnICON transient expression vectors pICH29921 and pICH31120 containing FGF21-F-IntN and IntC-Tf and **(C)** pTRAc transient expression vectors containing FGF21-F-Tf, FGF21-Tf-PLUS, FGF21-nTf338-PLUS, and nTf338-FGF21-PLUS in *N. benthamiana*. **(D)** The three-dimensional structures of different FGF21-Tf fusion proteins predicted by submitting the amino acid sequence to the RaptorX software (http://raptorx.uchicago.edu/). SP, signal peptide; F, furin cleavage site, RRKRSV; GS linker, flexible linker, (GGGGS)_3_; 2xHis, double-His_6_ tag; SEKDEL, ER-retention signal; IntN and IntC, DnaB intein fragments; PLUS, liver-targeting peptide circumsporozoite protein (CSP), aa 82–100; nTf338, N-terminal domain of Tf, aa 1–338.

The FGF21-Tf fusion constructs (Figures 1A–D and Supplementary Figure 1) were designed using the following elements: mature human FGF21 without signal peptide (SP) and including the point mutation Ser167Ala to eliminate the O-linked glycosylation site (Kharitonenkov et al., 2013), a GS flexible linker [(GGGGS)_3_] (Choi et al., 2014), a furin cleavage site (RRKRSV) (Duckert et al., 2004; Kwon et al., 2018), mature human Tf or the N-terminal domain of Tf (nTf338) (Mason et al., 1996), the N-terminal and C-terminal parts of the DnaB intein (Evans et al., 2000; Sun et al., 2001; Kempe et al., 2009), and the liver-targeting peptide PLUS of the circumsporozoite protein CSP (Lu et al., 2014; Ma et al., 2014, 2017; Taverner et al., 2020). The calreticulin SP of *Nicotiana plumbaginifolia* and the ER retention signal (SEKDEL) were used for targeting to the endoplasmic reticulum (ER). All constructs contained a C-terminal double His-tag for Immobilized Metal Ion Affinity Chromatography (IMAC) purification (Khan et al., 2006).

The amino acid sequences for the individual proteins (Figures 1A,B and Supplementary Figure 1) were assembled *in silico* and back-translated into a nucleotide sequence and codon-optimized for *N. tabacum* using the Eurofins gene optimizer software (Eurofins, Ebersberg, Germany). *Bam*HI/*Nru*I, *Bsa*I/*Bsa*I, and *Nco*I/*Xba*I restriction sites were added to the 5′ and 3′ end of the final nucleotide sequence to allow directional cloning into the p9U (DNA Cloning Service e.K., Hamburg, Germany), the MagnICON vectors pICH29921 and pICH31160 (Gleba et al., 2005; Marillonnet et al., 2005; Giritch et al., 2006) and the pTRAc vector (Maclean et al., 2007), respectively. The final nucleotide sequences were synthesized by Eurofins and delivered in pEX vectors from which they were transferred into the target vectors *via* restriction/ligation. The final vectors were verified by sequencing before introducing them into the *Agrobacterium tumefaciens* strains C58C1 (p9U), ICF320 (MagnICON) and GV3101:pMP90RK (pTRAc).

### Stable transformation of *Nicotiana tabacum*

*Nicotiana tabacum* cultivars VG and SL632 were used in this study. The seeds were surface sterilized in a saturated calcium hypochlorite solution with 0.1% (m v^–1^) Triton X-100 for 5 min. The seeds were rinsed with sterile distilled water several times and then germinated on Linsmaier and Skoog (LS) medium (Duchefa, Harleem, Netherands) supplemented with 30 g L^–1^ sucrose, 6.5 g L^–1^ plant agar and adjusted to pH 5.7. The plants were maintained at 24/22°C day/night temperature with a 16 h photoperiod. Subsequently, 4-week-old tobacco leaves were used for *Agrobacterium*-mediated transformation (Nausch et al., 2016). Regenerated shoots were selected on LS medium containing 0.1 g L^–1^ kanamycin and 0.5 g L^–1^ cefotaxim. Transgene integration was confirmed by PCR using FGF21- and Tf-specific primers (Supplementary Table 1).

### Plant cultivation in the greenhouse

Transgenic individuals were transferred from tissue culture 4 weeks after the last subculture directly into 5-L pots containing peat soil (Stender, Schermbeck, Germany). Plants were fertilized twice a week using 0.2% (m v^–1^) Hakaphos Blue (Compo Expert, Münster, Germany) or 0.1% (m v^–1^) Ferty 2 Mega (Nitsch & Sohn, Kreuztal, Germany).

### Transient transformation of *Nicotiana benthamiana*

The *N. benthamiana* plants were grown in the greenhouse until the age of 6−8 weeks in peat soil (Stender, Schermbeck, Germany) and fertilized with 0.2% (m v^–1^) Hakaphos Blue or 0.1% (m v^–1^) Ferty 2 Mega, in both cases with additional illumination (140 μmol s^–1^ m^–2^) to ensure a 16 h photoperiod. *A. tumefaciens* was cultivated either in lysogeny broth (LB) (Nausch et al., 2012b) or peptone agrobacterium medium (PAM) (Houdelet et al., 2017) containing antibiotics (MagnICON – 50 mg L^–1^ rifampicin and 50 mg L^–1^ kanamycin; pTRAc – 25 mg L^–1^ rifampicin, 25 mg L^–1^ kanamycin and 50 mg L^–1^ carbenicillin) for 24−48 h at 28°C in an orbital shaker (150 rpm) before dilution 1:100 in the same medium. After another 24−48 h of incubation at 28°C, the bacteria were centrifuged (2,800 × *g*, 15 min, 22°C) and resuspended in infiltration buffer [MagnICON – 10 mM *2*-(N-*morpholino*)ethanesulfonic acid (MES), 10 mM magnesium sulfate, pH 5.5; pTRAc – 0.5 g L^–1^ MS basal salts and 0.2 mM acetosyringone, pH 5.6] to a final OD_600*nm*_ of 0.1−0.5. For vacuum infiltration, *N. benthamiana* plants were submerged upside down into the bacterial suspension, a vacuum of 100 mbar applied for 2 min in a desiccator, and the plants then returned to normal growth conditions. *N. benthamiana* leaf material was harvested 10 days post-infection (dpi) for the MagnICON vectors and 5 dpi for the pTRAc vectors.

### Small-scale protein extraction

Each 100 mg seed or leaf material were homogenized in extraction buffer (25 mM sodium phosphate, 500 mM sodium chloride and 10 mM sodium bisulfite, pH 8.0) at a biomass/buffer ratio of 1:10 and 1:3, respectively, using a FastPrep-24 5G (MP Biomedicals, Irvine, CA, USA) and 0.5 g Zirconia/Silica beads for 3× at 8 Hz for 40 s with 30 s breaks on ice in between. The homogenate was clarified by 2 × centrifugation (13,520 × *g*, 20 min, 4°C) and the supernatant, containing the FGF21-transferrin fusion protein, collected.

### Large-scale protein extraction for immobilized metal ion affinity chromatography purification

The extraction and purification for IMAC was carried at 4°C. Each 800 g leaf material was homogenized in extraction buffer (25 mM sodium phosphate, 500 mM sodium chloride and 10 mM sodium bisulfite, pH 8.0) at a ratio of 1:3 biomass using an HR3655/00 Standmixer (Philips, Amsterdam, Netherlands) 3× for 30 s with 30 s breaks. In the standard protocol, the homogenate was initially clarified by a BP420 bag filter (Fuhr, Klein-Winternheim, Germany), then passed through a combination of K700 (6−15 μm nominal retention rating) and KS50 (1 μm nominal retention rating) depth filters (Pall Corporation, New York, NY, USA) or applied to 2 × ultracentrifugation (15,900 × *g*, 30 min, 4°C) before final sterile filtration using Sartopore 2 Capsule (pore size of 0.45 and 0.22 μM) (Sartorius, Göttingen, Germany). The clarified extract (free of cell debris, which remained in the filter cake), containing the FGF21-transferrin, was used for further purification.

His-tagged FGF21-transferrin was purified on an ÄTKA pure system (GE Healthcare, Chicago, IL, USA) by applying 4 L filtrated extract to a XP26/20 column packed with 58 mL IMAC Chelating Sepharose Fast Flow resin charged with Ni^2+^ ions. After washing with 5 column volume (CV) extraction buffer, the FGF21-transferrin fusion protein was eluted with 3 CV elution buffer (25 mM sodium phosphate, 500 mM sodium chloride and 150 mM imidazole, pH 7.6). A Vivaspin 15R centrifugal concentrator with a molecular weight cut off (MWCO) of 30 kDa (Sartorius, Göttingen, Germany) was used for buffer exchange into phosphate buffered saline (PBS) (137 mM sodium chloride, 2.7 mM potassium chloride, 12.5 mM disodium hydrogen phosphate, 2.0 mM potassium dihydrogen phosphate, pH 7.4).

### Protein quantification, Western blot, densitometric analysis, and ELISA

The total soluble protein (TSP) concentration in extracts and supernatants, including the FGF21-transferrin fusion protein and the host cell proteins (HCPs), was quantified *via* Pierce Coomassie Protein Assay-Kit (Thermo Fisher Scientific, Waltham, MA, USA).

For Western blot analysis, 100 μg or 10 μg TSP per sample were precipitated with trichloroacetic acid (TCA) and resuspended in NuPAGE LDS sample buffer, optionally supplemented with 0.5% (v v^–1^) β-mercaptoethanol and 100 mM dithiothreitol (DTT). Samples that were extracted with the NuPAGE LDS sample buffer were directly applied to Western blot analysis. The samples were denatured at 70°C for 10 min, separated on Invitrogen Novex NuPAGE 4−12% (m v^–1^) Bis-Tris protein gels (Thermo Fisher Scientific, Waltham, MA, USA) at 200 V at 22−24°C for 35 min, and transferred to Amersham Protran nitrocellulose membranes (VWR, Radnor, PA, USA) at 50 V at 22−24°C for 1 h using a Trans Blot Cell (Bio-Rad Laboratories, Hercules, CA, USA). The membrane was blocked with PBS containing 0.05% (m v^–1^) Tween-20 (PBST) and 5% (m v^–1^) skimmed milk powder for 1 h at 22−24°C. After washing 3× with PBST for 5 min, the membrane was probed at 4°C overnight with a polyclonal rabbit anti-His antibody (Hölzel Diagnostika Handels, Cologne, Germany) diluted 1:5,000 (0.1 mg L^–1^) or a polyclonal rabbit anti-FGF21 antibody (Dianova, Hamburg, Germany) diluted 1:5,000 (0.2 mg L^–1^) in Signal Boost ImmunoReaction Enhancer solution I (Merck, Darmstadt, Germany). Following another wash in PBST, the membrane was probed at 22−24°C for 1 h with a horseradish peroxidase (HRP)-conjugated goat anti-rabbit diluted 1:5,000 (0.06 mg L^–1^) in Signal Boost ImmunoReaction Enhancer solution II. After another wash with PBST, the signal was detected using the NBT/BCIP alkaline phosphatase system (Thermo Fisher Scientific, Waltham, MA, USA). Densitometric analysis of Western blot signals were conducted using the AIDA Image Analyzer analysis software (Elysia-raytest, Straubenhardt, Germany).

The FGF21 ELISA was carried out using the SimpleStep human FGF21 ELISA Kit (Abcam, Cambridge, UK) for both *in planta* and *in vivo* quantifications.

Intact FGF21-transferrin fusion protein accumulation was obtained through multiplying the ELISA-derived total product concentration by the fraction of intact FGF21-transferrin determined *via* anti-FGF21 Western blot analysis.

### Animals and oral delivery experimental setup

Oral delivery experiments were performed in adult FGF21^–/–^ mice generated previously (Ost et al., 2016) that were group-housed and allowed *ad libitum* access to food and water prior to experiment. Mice were maintained on a 12 h light/dark cycle. Prior to oral gavage, mice were individually housed and fasted for 16 h overnight. Since in previous studies (Camporez et al., 2013; Emanuelli et al., 2014), 1 mg kg^–1^ d^–1^ was applied to mice to obtain an FGF21-mediated stimulation, the FGF21-mice received a 0.5 mL bolus of partially purified nTf338-FGF21-PLUS, i.e., freeze-dried FGF21-transferrin that contained a substantial fraction of HCPs, dissolved in water to a concentration of 50 μg L^–1^ FGF21 and 44 g L^–1^ HCP derived from IMAC purification of mock-infiltrated *N. benthamiana* plants. The control mice were gavaged with HCP only. After 4 h, mice were euthanized with an overdose of Ketamine/Xylazine and Isoflurane and subsequent heart puncture. Following blood collection, tissues were isolated and snap frozen in liquid nitrogen. All experiments were approved by the Ethics Committee of the Ministry for Environment, Health and Consumer Protection of Brandenburg, Germany (approval no. 2347-9-2020).

Plasma samples for the FGF21 ELISA were undiluted, and 30 mg ground tissue was homogenized in tissue extraction buffer [10 mM Tris-HCl and 0.02% (m v^–1^) Triton X-100, pH 7.4] at a ratio of 1:3 tissue mass using a TissueLyser LT (Qiagen GmbH, Germany) for 3 min at 50 Hz, and subsequent freeze-thaw (−20°C overnight) and centrifugation (23,000 × *g*, 20 min, 4°C).

mRNA analysis was performed as previously described (Weitkunat et al., 2016). Briefly, total RNA was extracted from 30 mg ground tissue using peqGOLD TriFast reagent (VWR, Germany). Following DNase treatment, cDNA synthesis was performed with 1 μg RNA to a final concentration of 5 mg L^–1^ according to suppliers’ protocol (LunaScript RT SuperMix, NEB, Germany). Gene expression was calculated as ddCT, using *B2m* as the normalizer gene (Supplementary Table 1), where the control group was set to a value of 1.

### Three-dimensional structure analysis

The three-dimensional structure analysis of the FGF21-transferrin fusion proteins was conducted by submitting the amino acid sequence to the RaptorX software^5^ (Källberg et al., 2012).

### Statistical methods

Statistical analysis was performed in Origin 2020b (OriginLab, Northampton, MA, USA). The Shapiro–Wilk- and *F*-test of OriginPro was used to ensure normal distribution and equal variance of the dataset, followed by a univariate ANOVA (including the *post hoc* Bonferroni test). *p*-Values of 0.05 (* and letters), 0.01 (^**^) and 0.001 (^***^) were considered to indicate significant, strongly significant and highly significant differences as indicated in the individual experiments. There were no non-normal distributed data.

## Results

### Pruning increases tobacco seed yield in the greenhouse

Biopharmaceutical safety is often ensured by the production in contained environments, e.g., greenhouses in case of plants (Holtz et al., 2015). The corresponding cultivation conditions have been well established for the cultivar *N. tabacum* VG (Nausch et al., 2016), which is why we selected this cultivar in this study. The cultivar *N. tabacum* SL632 was also included in this study (Sunchem NL, Amsterdam, Netherlands) because this cultivar is nicotine-free and has been bred to a high seed yield of >9 tons SDM per hectare and year under open field conditions (Sunchem NL, Amsterdam, Netherlands^4^). However, SL632 has not yet been used for seed production in a greenhouse setting and thus required cultivation strategy adaptation to increase seed productivity.

Under standard cultivation conditions (uncut), both tobacco cultivars grew up to a height of 1.98−2.20 m, and when harvested after 136 and 107 days, SL632 yielded ∼43 g SDM per plant compared to 18 g SDM per plant in case of VG (Table 1). Noteworthy, the higher seed biomass of SL632 resulted from both an increased seed number and mass, e.g., a thousand SDM of ∼0.107 g (SL632) compared to ∼0.082 g (VG) (Supplementary Table 2). We reasoned that seed yields per plant can be increased through additional side branches with inflorescences. The latter are induced if the apical dominance of the main shoot is suppressed, for example by removing the tip of the main shoot either by cutting it at a height of ∼1 m (1× cut) or by removing the top ∼0.2 m (1× top, Table 1). Both approaches delayed the harvest time by 10−20% but at the same time generated up to 30% more side branches, which increased the seed yield by ∼50% in case of SL632 and ∼100% in case of VG (Table 1). We assume that the 1× cut effect on VG was more pronounced than for SL632 because VG formed less side branches in the uncut state than SL632 (Table 1). Nevertheless, the absolute seed yield of SL632 was approximately twofold higher than that of VG, and the production costs per seed biomass were lowest for cutting of SL632 with 1.64 € g^–1^ (Table 2).

**TABLE 1:** Plant traits of *N. tabacum* SL632 and VG under different cultivation strategies in the greenhouse.

*N. tabacum* cultivar	Parameter	Description	Unit	Uncut	1× top	1× cut
SL632	1	Treatment time	dps	na	62.0 ± 0.9 (a)	57.8 ± 1.0 (b)
	2	Flowering of main stem	dps	61.8 ± 0.8	na	na
	3	Side branches with inflorescence	–	21.5 ± 4.7 (a)	28.4 ± 3.4 (b)	26.7 ± 3.4 (b)
	4	Side branch inflorescence flowering time after seeding	dps	61.8 ± 0.8 (a)	72.3 ± 3.2 (b)	82.0 ± 4.9 (c)
	5	Plant height	cm	219.5 ± 14.1 (a)	206.4 ± 11.5 (a)	206.6 ± 12.6 (a)
	6	Seed harvest	dps	135.6 ± 11.0 (a)	140.8 ± 12.2 (ab)	149.3 ± 6.2 (b)
	7	Total SDM per plant	g	42.7 ± 4.7 (a)	61.4 ± 6.6 (b)	67.9 ± 11.9 (b)
	8	Seed productivity	g d^–1^	∼0.315	∼0.436	∼0.455
VG	1	Treatment time	dps	na	66.3 ± 1.6 (a)	62.0 ± 1.5 (b)
	2	Flowering of main stem	dps	65.0 ± 1.0	na	na
	3	Side branches with inflorescence	–	18.9 ± 2.4 (ab)	15.4 ± 3.0 (a)	22.1 ± 5.6 (b)
	4	Side branch inflorescence flowering time after seeding	dps	65.0 ± 1.0 (a)	72.2 ± 6.7 (b)	86.3 ± 5.4 (c)
	5	Plant height	cm	197.8 ± 18.7 (a)	221.3 ± 17.5 (b)	217.6 ± 11.6 (b)
	6	Seed harvest	dps	107.4 ± 0.5 (a)	117.1 ± 4.1 (b)	127.8 ± 3.9 (c)
	7	Total SDM per plant	g	18.0 ± 4.9 (a)	24.4 ± 4.4 (a)	43.1 ± 8.7 (b)
	8	Seed productivity	g d^–1^	∼0.168	∼0.208	∼0.337

Numbers represent mean ± SD. Significance was calculated by one-way ANOVA with Bonferroni correction with an alpha threshold of 0.05 and significance groups indicated by letters (a, b, and c). The number of biologic replicates was *n* = 12 (parameter 1−6) and *n* = 4−5 (parameter 7). a, b, c – significance groups (*p* < 0.05). SDM, seed dry mass; dps, days post seeding; na, not applicable.

**TABLE 2:** Operating expenses (OPEX) for tobacco seed and leaf biomass production per plant in the greenhouse *via* different cultivation strategies.

Tobacco cultivar	Biomass type	Cultivation strategy	Seed and leaf production* (week)	Working hours (h)	Labor costs (€)	Consumable costs (€)	Total costs per plant (€)	SDM/LFM yield per plant (g)	Costs per SDM/LFM (€ g^–^^1^)
SL632	Transgenic seed	Uncut	28	1.0	89.9	1.4	91.3	42.7	2.14
	Transgenic seed	1× Top	29	1.2	107.7	1.4	109.1	61.4	1.78
	Transgenic seed	1× Cut	30	1.3	109.9	1.4	111.3	67.9	1.64
	Transgenic leaf	Uncut	6	0.7	62.6	1.4	64.0	340.3	0.19
VG	Transgenic seed	Uncut	24	1.0	89.9	1.4	91.3	18.0	5.07
	Transgenic seed	1× top	25	1.2	107.7	1.4	109.1	24.4	4.47
	Transgenic seed	1× cut	27	1.3	109.9	1.4	111.3	43.1	2.58
	Transgenic leaf	Uncut	6	0.7	62.6	1.4	64.0	464.2	0.14
Nb	Transient leaf	Uncut	6	0.2	20.6	0.3	20.8	155.0	0.13

Costs per plant were calculated per batch based on a batch size of 72 plants for *N. tabacum* cv. SL632 (SL632) and VG (VG) and 400 plants for *N. benthamiana* (Nb). Labor costs were those of a technical assistant in Germany (2021; 87 € h^–1^; full overhead calculation). Consumables covered water, soil, fertilizer, and energy expenses. Nb, *N. benthamiana*; LFM, leaf fresh mass; SDM, seed dry mass; SL632, *N. tabacum* cv. SL632; VG, *N. tabacum* cv. VG. *Seed production includes plant growth until seed harvest (Table 1) and seed drying 8 weeks.

In contrast to seeds, the production of leaf biomass is well established for tobacco (Conley et al., 2011; Nausch et al., 2016) and *N. benthamiana* used for transient expression (Holtz et al., 2015; Buyel et al., 2017; Huebbers and Buyel, 2021). In this case, leaves can be harvested after ∼6 weeks compared to the ∼21 weeks for seeds (without seed drying) required for SL632 after cutting. Typical LFM yields were ∼340, ∼460, and ∼155 g per plant for SL632, VG and *N. benthamiana* with production costs of 0.19 € g^–1^ (SL632), 0.14 € g^–1^ (VG) and 0.13 € g^–1^ (*N. benthamiana*) (Table 2). Moreover, due to the 50% lower staff costs, leaf biomass production averaged only 10% of the total costs of seed production (Table 2). In this context semi-automated cultivation systems might be of interest, which can reduce the cultivation costs by more than 90% (Huebbers and Buyel, 2021).

### Accumulation of FGF21-F-Tf in tobacco seeds was limited by *in planta* degradation

For oral delivery of FGF21 *via* tobacco seeds, we used mature human FGF21 without O-glycosylation site to obtain a homogenous product as previously described (Kharitonenkov et al., 2013) and fused the coding region of mature human FGF21 to Tf, which mediates the transfer from the intestine to the portal vein *via* the Tf-receptor (Figurea 1A–D and Supplementary Figure 1; Choi et al., 2014). We included a GS-linker to avoid steric hindrance between FGF21 and Tf, and a furin cleavage site, yielding FGF21-F-Tf (Wilbers et al., 2016). The furin cleavage site does not occur in plants but should facilitate the release of FGF21 from Tf during uptake in the enterocytes of the intestine. Since Tf requires disulfide bonds for authentic folding (Mason et al., 1996), the FGF21-F-Tf fusion protein was targeted to the ER. Therefore, we substituted the endogenous N-terminal SP of FGF21 with that of calreticulin of *N. plumbaginifolia*, because the plant SP can improve the accumulation level compared to the native one in plants (Shaaltiel et al., 2007; De Marchis et al., 2011). We also added the ER retention signal SEKDEL to the C-terminus of Tf because the ER displays a lower proteolytic activity compared to the apoplast (Benchabane et al., 2008), which might favor the accumulation level. Finally, in order to compare accumulation levels in seeds to leaves, and we employed the constitutive 35S CaMV expression cassette (Figure 1A).

**FIGURE 2:**
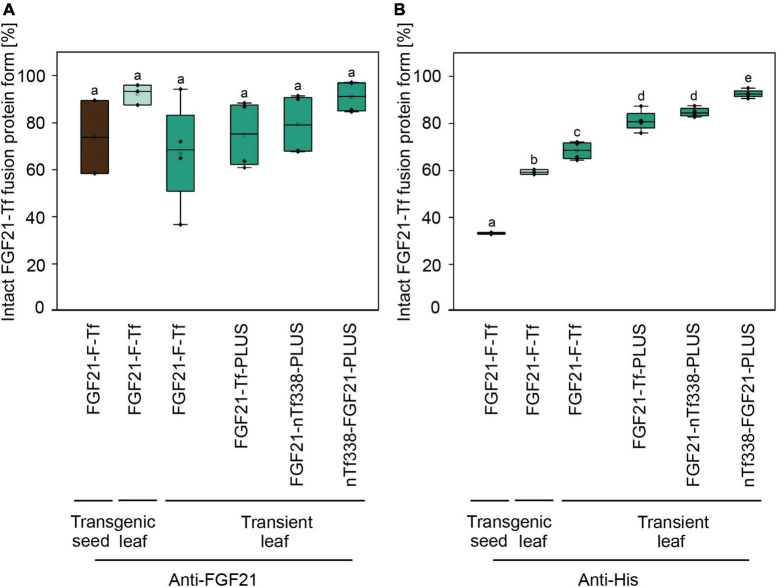
Degree of degradation during extraction of optimized FGF21-transferrin fusion proteins in *N. tabacum* SL632 (SL1-1) and in *N. benthamiana*. Relative fraction of the intact FGF21-Tf fusion proteins in plant extracts, detected by **(A)** anti-FGF21 and **(B)** anti-His Western blots and band intensities quantified using the AIDA Image Analyzer analysis software. *n* = 3, biological replicates.

We first expressed the fusion protein transiently in *N. benthamiana* using the MagnICON system (Figure 1B) (Marillonnet et al., 2004, 2005; Giritch et al., 2006). FGF21 and Tf were co-expressed as separate proteins that were post-translationally fused *via* an intein tag (Evans et al., 2000; Sun et al., 2001; Kempe et al., 2009) as the insert size of the viral replicon was limited to 2 kbp. FGF21 levels measured with an ELISA indicated a yield of 2.1 mg kg^–1^ LFM (Table 3). However, while the anti-FGF21 Western blot showed the expected FGF21-F-Tf full-length size (∼100 kDa), the anti-His Western blot revealed the presence of free Tf (∼80 kDa) (Supplementary Figure 2A). To identify whether this observation was due to imperfect coupling of Tf to FGF21 *via* the intein tag or a result of proteolytic degradation, a pTRAc vector was used for expression, since this vector facilitated the expression of FGF21-F-Tf as a single in-frame fusion protein (Figure 1C). This approach yielded 1.2 mg kg^–1^ LFM according to the anti-FGF21-ELISA (Table 3) and degradation products were detected in addition to the intact fusion protein both in anti-FGF21 (Figure 2A and Supplementary Figure 2A) and anti-His (Figure 2B and Supplementary Figure 2A) Western blots. Based on a densitometric analysis, ∼33% of FGF21-F-Tf was degraded in anti-FGF21 Western blot and ∼32% in anti-His Western blot, reducing the accumulation level of intact FGF21-F-Tf to 0.8 and 0.9 mg kg^–1^ LFM (Table 3, Figures 2A,B, and Supplementary Figure 2A). Degraded product was found even when extracting FGF21-F-Tf in denaturing LDS sample buffer, suggesting that degradation occurred *in planta* and not during the extraction process (Supplementary Figure 2B). Nevertheless, we proceeded to the generation of transgenic plants with the FGF21-F-Tf construct (Figure 1A) because others studies indicated that seeds are a suitable platform to produce recombinant proteins that are susceptible to proteolytic degradation (Yao et al., 2015).

**TABLE 3:** FGF21 accumulation level of FGF21-transferrin fusion constructs in crude extract.

Plant	Type*^a^*	Vector		Fusion protein (mg kg^–^^1^ biomass)	Total FGF21 fusion protein in crude extract (mg kg^–^^1^ TSP)	Intact FGF21 fusion protein in crude extract		Biological replicates	Biomass (kg m^–^^2^ a^–^^1^)	Intact FGF21 fusion protein yield (mg m^–^^2^ a^–^^1^)
						Anti-FGF21 WB (mg kg^–^^1^ biomass)	Anti-His WB (mg kg^–^^1^ biomass)			
*N. tabacum* SL632	Transgenic seed	p9U	FGF21-F-Tf	9.1 ± 0.3	63.6 ± 2.1	6.7 ± 0.2	2.2 ± 0.1	2	0.38	2.55
*N. tabacum* SL632	Transgenic leaf	p9U	FGF21-F-Tf	6.1 ± 0.2	68.5 ± 1.8	5.6 ± 0.2	3.3 ± 0.1	2	8.19	45.86
*N. tabacum* VG	Transgenic seed	p9U	FGF21-F-Tf	8.7 ± 0.0	84.2 ± 0.3	6.4 ± 0.0	2.1 ± 0.0	2	0.24	1.54
*N. tabacum* VG	Transgenic leaf	p9U	FGF21-F-Tf	2.4 ± 0.0	105.5 ± 1.4	2.2 ± 0.0	2.0 ± 0.0	2	11.17	24.57
*N. benthamiana*	Transient leaf	MagnICON	FGF21-F-Tf	2.1 ± 0.0	115.7 ± 1.7	2.1 ± 0.0	0.0 ± 0.0	2	11.94	25.07
		pTRAc	FGF21-F-Tf	1.2 ± 0.3	151.6 ± 51.4	0.8 ± 0.2	0.9 ± 0.1	3	11.94	9.55
		pTRAc	FGF21-Tf-PLUS	2.1 ± 0.7	446.9 ± 106.6	1.6 ± 0.5	1.3 ± 0.4	3	11.94	19.10
		pTRAc	FGF21-nTf338-PLUS	2.0 ± 0.6	427.9 ± 132.2	1.6 ± 0.5	1.3 ± 0.4	3	11.94	19.10
		pTRAc	nTf338-FGF21-PLUS	2.3 ± 0.6	361.1 ± 136.3	2.1 ± 0.5	1.9 ± 0.5	3	11.94	25.07

Numbers represent mean ± SD. Total FGF21 fusion protein was determined by the FGF21 ELISA and intact FGF21 fusion protein was obtained by correcting ELISA data by anti-FGF21 and anti-His Western blot analysis and estimation of the relative amount of intact fusion protein and cleaved FGF21. Biomass refers to leaf fresh mass (LFM) or seed dry mass (SDM), which were calculated with the help of the data shown in Table 2.

^a^Number for transgenic plants refer to seeds of the T1 generation and leaves of the T2 generation. WB, Western blot.

When cultivating SL632 in tissue culture before the transformation, the first SL632 plants started to flower after the second or third passage (with 3 weeks per passage), which limited the vegetative propagation to six passages. On the other hand, VG flowered only occasionally, was propagated for at least 19 passages and provided almost twice the number of positive transformants (Supplementary Tables 3, 4). Noteworthy, in VG the GFP control yielded twofold more positive transformants compared to the FGF21-F-Tf, indicating a potential negative impact on the cell viability (Supplementary Table 4). However, the T0 seed viability was equal to the control (Supplementary Table 5), which contradicts to that assumption.

**FIGURE 3:**
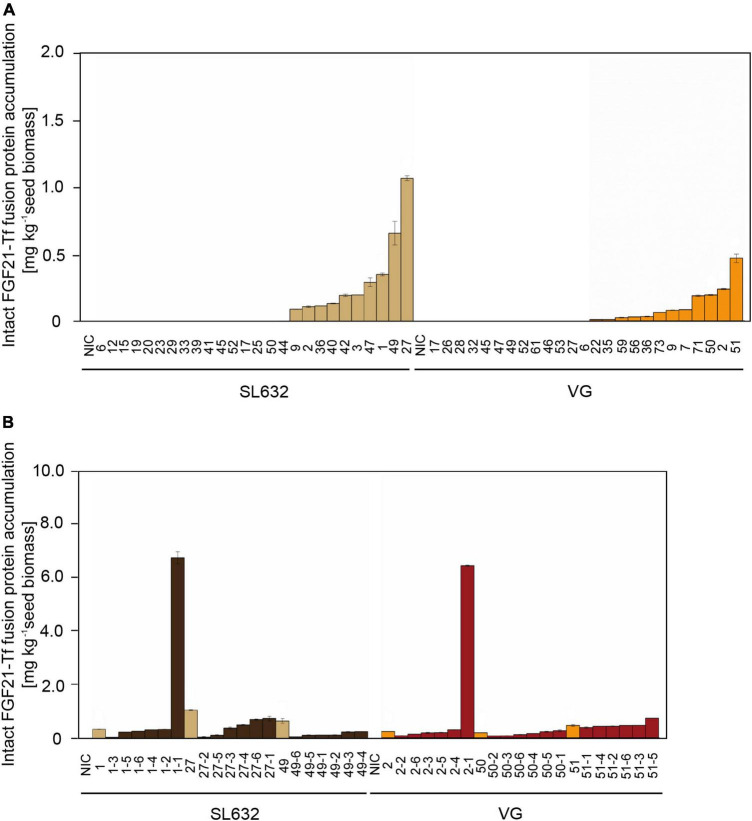
Intact FGF21-F-Tf fusion protein yield in seeds of T0 and T1 transformants in *N. tabacum* SL632 and VG. **(A)** Intact FGF21-F-Tf fusion protein accumulation in seeds of T0 transformants. **(B)** Intact FGF21-F-Tf fusion protein accumulation in seeds of T1 descendants of selected T0 transformants 1, 27, and 49 of SL632 and 2, 50, and 51 of VG. Intact FGF21-F-Tf fusion protein accumulation was obtained through multiplying the ELISA-derived total product concentration by the fraction of intact FGF21-F-Tf determined *via* anti-FGF21 Western blot analysis. *n* = 2, biological replicates, i.e., different leave samples from the same plant. NIC, near-isogenic control plants.

While the highest FGF21-F-Tf accumulation in seeds of the T0 transformant of SL632 was 2.2-fold higher compared to VG (Figure 3A), the maximal FGF21-F-Tf yield was similar with ∼6.5 mg kg^–1^ SDM in seeds of the T1 descendants for both cultivars (Figure 3B and Table 3). This complies with previous studies in which the expression of C5a and IL6, produced in seeds and leaves of transgenic tobacco, increased by about an order of magnitude from T0 to T1 (Nausch et al., 2012a,b). However, the increase in the FGF21-F-Tf was only observed in each one T1 individual of SL632 and VG, while in the other T1 plants the yield was similar or lower compared to the T0 transformant.

Unexpectedly, FGF21-F-Tf degradation was even higher in transgenic seeds, reaching ∼67% in the anti-His Western blot (Figure 2 and Supplementary Figure 3), and might be the reason for the low yield of intact FGF21-F-Tf of ∼6.5 mg kg^–1^ SDM in both cultivars (Table 3). Noteworthy, FGF21-F-Tf degradation in transgenic leaves of the same plants was substantially lower with 41% (Figure 2 and Supplementary Figure 3), and intact FGF21-F-Tf accumulated at 5.6 (SL632) and 2.2 (VG) mg kg^–1^ LFM (Table 3).

However, when using a denaturing LDS sample buffer instead of the extraction buffer (Supplementary Figure 3), the ratio of intact to degraded FGF21-F-Tf was similar in leaves but higher in seeds. This suggested that not all recombinant protein was extracted from seeds and that a substantial fraction of intact FGF21-F-Tf might be deposited in insoluble protein bodies or protein storage vacuoles.

Nevertheless, only the soluble FGF21-F-Tf might be relevant for the oral application. Accordingly, when combining the accumulation levels of soluble FGF21-F-Tf with the biomass productivity data (Tables 1, 2), yields of intact FGF21-F-Tf were 18-fold (SL632) and 16-fold (VG) higher in leaves compared to seeds with a productivity of 45.86 (SL632) and 24.57 (VG) mg m^–2^ a^–1^ in leaves compared to 2.55 (SL632) and 1.54 (VG) mg m^–2^ a^–1^ in seeds (Table 3).

### Fusion protein degradation was reduced by furin cleavage site removal and truncation of transferrin

We modified the fusion protein to minimize degradation and to increase the FGF21 yield, and tested the new fusion proteins *via* transient expression in *N. benthamiana* (Figure 1C). We analyzed the structures of all fusion protein variants *in silico*, by submitting the amino acid sequence to the RaptorX software (see text footnote 5), to identify potential steric hindrances prior to cloning, but did not observe any problems (Figure 1D). First, we removed the furin site from the fusion protein to rule out unintended degradation *in planta*. Because this modification may increase serum half-life of FGF21, triggering deleterious side effects (Talukdar et al., 2016), we included the PLUS peptide that mediates exclusive uptake by liver cells (Lu et al., 2014; Ma et al., 2014, 2017; Taverner et al., 2020). The resulting FGF21-Tf-PLUS construct (Figure 1) accumulated to 1.6 mg kg^–1^ LFM, which was twice the concentration of FGF21-F-Tf (Table 3), and degradation was reduced from 32 to 19% (Figure 2).

We also reasoned that the large size of the fusion protein of >100 kDa may provide unnecessary protease cleavage sites and could potentially limit accumulation, which has been reported for other fusion proteins produced in transgenic tobacco (Phan et al., 2014). Using only the first 338 n-terminal amino acids (nTf338), which mediate Tf receptor binding (Mason et al., 1996), did not increase the accumulation of the resulting FGF21-nTf3338-PLUS fusion protein but reduced degradation to 15% (Figure 2 and Table 3). Changing the domain sequence (nTf338-FGF21-PLUS) lowered degradation to 7%, while the accumulation of intact fusion protein increased to 2.1 or 5.8 mg kg^–1^ LFM when only non-senescent leaves were processed (Tables 3, 4).

**TABLE 4:** Comparison of purification process performance for nTf338-FGF21-PLUS extracted from non-senescent, transiently transformed *N. benthamian*a leaves, using filtration or centrifugation as the major clarification step.

Method	Depth filtration^a^				Ultracentrifugation^b^			
Process stage	Overall recovery (%)	Step recovery (%)	Yield (μ g kg^–^^1^ biomass)	Purity (%)	Overall recovery (%)	Step recovery (%)	Yield (μ g kg^–^^1^ biomass)	Purity (%)
Homogenate	100.00	–	2,930.1 ± 628.9	0.05 ± 0.02	100.00	–	5,779.0 ± 119.6	0.05 ± 0.00
Bag filtrate	76.3 ± 0.9	76.3 ± 0.9	2,234.5 ± 460.9	0.06 ± 0.03	55.5 ± 2.9	55.5 ± 2.9	3,206.7 ± 236.4	0.04 ± 0.00
Clarification (centrifugation or filtration)	4.2 ± 2.0	5.5 ± 2.6	121.3 ± 52.2	0.01 ± 0.00	52.5 ± 2.4	94.7 ± 0.7	3,035.3 ± 200.5	0.03 ± 0.00
Sterile filtrate	3.6 ± 1.7	85.5 ± 4.9	104.5 ± 46.1	0.01 ± 0.00	36.6 ± 0.4	69.9 ± 4.0	2,116.7 ± 19.5	0.02 ± 0.00
IMAC flow through	2.8 ± 1.5	76.8 ± 11.0	82.4 ± 43.0	0.04 ± 0.02	35.2 ± 0.4	96.1 ± 0.1	2,033.2 ± 19.9	0.08 ± 0.01
IMAC wash	2.7 ± 1.5	95.8 ± 2.9	79.7 ± 42.6	0.06 ± 0.02	34.9 ± 0.4	99.1 ± 0.0	2,014.5 ± 20.1	0.08 ± 0.01
IMAC elution	2.3 ± 1.4	84.4 ± 11.2	58.7 ± 39.8	0.14 ± 0.04	31.7 ± 0.3	90.9 ± 0.0	1,324.8 ± 8.9	0.19 ± 0.00

Yield and purity were determined by FGF21-ELISA and Bradford assay. ^a^*n* = 3; ^b^*n* = 2. IMAC, immobilized metal-ion affinity chromatography.

However, the higher accumulation level might not only result from an increased stability/reduced degradation of the fusion proteins, but also from an increased expression for which the mRNA level can be an indicator. The analysis of the latter and testing different codon-optimized construct variants for the same fusion protein might be done in the future to further improve the accumulation level of nTf338-FGF21-PLUS.

In this context might replicating geminiviral vectors such as the BeYDV (Diamos et al., 2020) be used as alternative to the non-replicating pTRAc vector, used in the study, since the geminiviral DNA replicon does not have an insert size limitation as well and might increase the transgene copy number, transcription rate and mRNA level.

Nevertheless, based on the sevenfold increase in the accumulation of FGF21-F-Tf, when switching from transient to transgenic expression in leaves (Table 3), we assume that nTf338-FGF21-PLUS will exhibit a similar increase once stably transformed into tobacco plants.

### Depth filtration limited the recovery of nTf338-FGF21-PLUS

The accumulation levels obtained in transiently and stably transformed plants was not sufficient for animal feeding studies for which the concentration needed to be >50 mg kg^–1^ biomass in order to be able to apply 1 mg kg^–1^ d^–1^ of nTf338-FGF21-PLUS per mice like in previous studies (Camporez et al., 2013; Emanuelli et al., 2014). Hence, we purified transiently expressed nTf338-FGF21-PLUS from *N. benthamiana* leaves, using a previously reported protocol (Menzel et al., 2018; Opdensteinen et al., 2021a,b). Whereas the step recovery average >75% for most process steps, it was limited to ∼5% for the depth filtration (Table 4). Using flocculants in combination with wide-pore filters or filters that are free of diatomaceous earth can help to resolve this bottleneck in the future (Buyel and Fischer, 2014; Buyel et al., 2015). However, even though knowing that this operation can be difficult to scale up and may need replacement in a large-scale process, in this proof-of-concept study, we replaced depth filtration by ultracentrifugation, yielding a step recovery of ∼95%. This indicated that some nTf338-FGF21-PLUS may be attached to particulate matter, which is retained by the filters. Adding low concentrations of detergents, such as Triton X-100, may help to increase the recovery in the future. Nevertheless, the overall recovery of the centrifugation-based process was ∼32% corresponding to ∼1.3 mg kg^–1^ LFM (Table 4).

Irrespective of the method, the purity remained below 0.2% FGF21 relative to the TSP after IMAC (Table 4 and Supplementary Figure 4). This was an unusual result and the low initial nTf338-FGF21-PLUS concentration was a likely reason for unspecific HCP binding to the resin, reducing the product purity due to co-elution. In the future, the purification might be simplified and/or improved by incorporating alternative HCP precipitation strategies such as heat or pH (Buyel et al., 2016; Opdensteinen et al., 2021a).

Because previous animal feeding trials have successfully used freeze-dried, re-suspended leaf material, crude extracts or partially purified recombinant protein, e.g., CTB-Ex-4 or Ex-4-Tf, without any obvious negative impact of the impurities (Kwon et al., 2013; Choi et al., 2014; Phan et al., 2020), we proceeded with the partially purified nTf338-FGF21-PLUS

### nTf338-FGF21-PLUS was bioactive and enabled selective FGF21 uptake into the liver without impacting bioactivity

**FIGURE 4:**
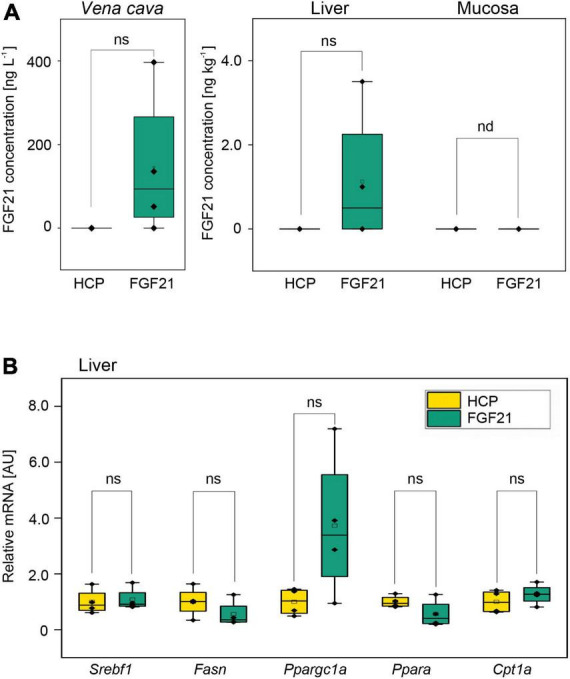
The mean bioavailability and bioactivity of the purified fusion protein nTf338-FGF21-PLUS in an *in vivo* animal bolus feeding trial. FGF21^– /–^ knockout mice were starved for 16 h and gavaged with a 0.5 mL bolus of partially purified nTf338-FGF21-PLUS dissolved in water to a concentration of 50 μg L^– 1^ FGF21 and 44 g L^– 1^ HCP (FGF21 group) or HCP only (HCP group). After 4 h, mice were sacrificed. **(A)** FGF21 concentration in the *Vena cava*, liver and mucosa of *via* FGF21-ELISA. **(B)** mRNA expression levels of the gene *Srebf1*, *Fasn*, *Ppargc1a*, *Ppara*, and *Cpt1a*, determined in liver tissue *via* qPCR (primer are listed in Supplementary Table 1). Numbers represent mean ± SD. Significance was calculated by pair-sample *t*-test with *p < 0.05, **p < 0.01, and ***p < 0.001. *n* = 4, biological replicates. ns, not significant; nd, not detectable.

The bioavailability and bioactivity of partially purified nTf338-FGF21-PLUS was tested *in vivo* using FGF21^–/–^ knockout mice. When nTf338-FGF21-PLUS was dissolved in water and orally gavaged (25 μg in 0.5 ml per mouse), FGF21 could be detected by ELISA in plasma obtained from the *Vena cava* in three and in liver homogenates in two out of four treated mice after 4 h (Figure 4A and Supplementary Figure 5A). In none of the supplemented mice could FGF21 be detected in the intestinal mucosa suggestive of an efficient transfer from the intestine to the liver.

Gene expression analysis of the liver revealed that the expression of the master regulator of fatty acid synthesis, the sterol regulatory element binding transcription factor 1(*Srebf1)*, was unaffected by FGF21, while its downstream target, the fatty acid synthase (*Fasn*) (Horton et al., 2002), seemed to be downregulated (Figure 4B and Supplementary Figure 5B). Unexpectedly, the expression of the regulator of fatty acid oxidation *Ppara* (peroxisome proliferator-activated receptor alpha) seemed also to be downregulated, while the expression of one of the targets of *Ppara*, *Cpt1a* (carnitine palmitoyltransferase 1a), a key enzyme in β-oxidation, (Ventura-Clapier et al., 2008) was unchanged (Figure 4B and Supplementary Figure 5B). However, the expression of a master regulator of mitochondrial biogenesis *Ppargc1a*, the gene encoding peroxisome proliferator-activated receptor gamma coactivator 1-alpha (PGC-1α) (Jornayvaz and Shulman, 2010), even though not significant, seemed to be upregulated as expected. These dara indicated that orally deliverd nTf338-FGF21-PLUS can affect hepatic lipid metabolism.

## Discussion

We showed that a plant-derived FGF21-transferrin fusion protein can be used for oral delivery of FGF21 as a potential therapeutic of NASH and provide an initial comparison of different production options and associated costs in transgenic seeds and leaves which both might be used for oral delivery.

We demonstrated for SL632 that the tobacco seed yield can be improved by pruning to 380 g m^–2^ a^–1^ in a greenhouse with manufacturing costs of 1.64 € g^–1^ SDM compared to 8,193 g m^–2^ a^–1^ and costs of 0.19 € g^–1^ LFM for leaves.

When comparing SL632 seeds and VG leaves as production platforms, the biomass productivity per unit area and time was up to 30-fold higher (Table 3) and the production costs up to 15-fold lower for leaves (Table 2). However, ∼98% of the costs were labor costs and assuming that these can be reduced by 90% when using semi-automated greenhouse facility (Huebbers and Buyel, 2021), the overall seed and leave production costs might be below 0.05 and 0.01 € g^–1^ (Nandi et al., 2005). Based on a recent clinical study with rice seeds, 6 g can be sufficient for oral vaccination (Yuki et al., 2021) and costs of <0.50 € per dose would also be affordable for developing countries and thus drastically lower compared to therapeutics, containing purified recombinant protein from transiently transformed leaves of up to 500 € per dosage (Tusé et al., 2014).

Independent from that, we produced an intact, bioactive FGF21-F-Tf fusion protein, accumulating to 6.7 and 5.6 mg kg^–1^ in transgenic tobacco seeds and leaves respectively, whereas transient expression in *N. benthamiana* leaves generated up to 2.1 mg kg^–1^. The yield of FGF21-F-Tf in seeds was 22-fold higher compared to recombinant IL6 for which the constitutive 35S CaMV expression cassette has been used as well but substantially lower compared to other proteins that were expressed with a seed-specific promotor/terminator in tobacco seeds at accumulation levels of 190−6,500 mg kg^–1^ SDM (Supplementary Table 6; Fiedler and Conrad, 1995; Kusnadi et al., 1998; De Jaeger et al., 2002; Cheung et al., 2009; Zimmermann et al., 2009; Zhang et al., 2010; Nausch et al., 2012b; Hensel et al., 2015; Hernandez-Velazquez et al., 2015; Weichert et al., 2016; Ceballo et al., 2017; Queiroz et al., 2019). Therefore, using a seed-specific expression cassette might increase FGF21-Tf fusion protein yields in the future.

However, the accumulation of FGF21-F-Tf was limited by proteolytic degradation (Figure 2 and Supplementary Figures 2, 3) and the removal of the furin cleavage site reduced the amount of degradation products. On the one hand, this indicated that even though the mammalian protease furin does not occur in plants (Wilbers et al., 2016), furin-like plant proteases such as Kex2p may cleave furin sites (Kinal et al., 1995). This observation contradicts previous reports in which recombinant proteins, containing a furin cleavage site, did not exhibit degradation in plants (Verma et al., 2010; Boyhan and Daniell, 2011; Kang et al., 2018; Kwon et al., 2018; Mamedov et al., 2019; Margolin et al., 2020). On the other hand, degradation may not be linked to the furin cleavage site itself but to the GS-linker region surrounding the furin site as observed for other fusion proteins (Benchabane et al., 2008) and as reported for the GS-linker (Chen et al., 2012). Such a non-exclusive cleavage is in agreement with our finding that removing the furin cleavage site reduced but did not completely prevent FGF21-transferrin degradation (Figure 2 and Supplementary Figure 2). In this respect, other linkers could be tested in the future to increase FGF21-transferrin fusion protein stability, e.g., a rigid EA-linker (Supplementary Table 7; Amet et al., 2009; Kim et al., 2010; Fu et al., 2011; Wang et al., 2011, 2014; Chen et al., 2012, 2018; Choi et al., 2014; Liu et al., 2020). However, a linker may not be necessary at all as we showed by using the liver-targeting PLUS peptide (Lu et al., 2014; Ma et al., 2014, 2017; Taverner et al., 2020). In a next step, the accumulation of this optimized construct in combination with a seed-specific expression cassette should be tested in transgenic seeds to obtain accumulation levels sufficient for oral delivery.

Alternatively to tobacco, other crops such as rice, barley, maize or pea and soybean can be used for the production of FGF21-transferrin fusion proteins as well (Supplementary Table 6), since these crops yield similar quantities of recombinant protein. For example, Tf yielded 10.00 g kg^–1^ in rice seeds (Supplementary Table 6). In addition, commercial, semi-automated greenhouse facilities have been established for rice (Ventria Bioscience, Junction City, KS, USA) (see text footnote 1) and barley (ORF Genetics, Kópavogur, Island) (see text footnote 2).

Importantly, more FGF21-F-Tf degradation was observed in transgenic seeds (67%) than in transgenic leaves (41%) (Figure 2), indicating that, opposed to the common notion (Benchabane et al., 2008), protein expression in seeds may not always be advantageous in terms of product stability and accumulation. The reduced degradation of FGF21-F-Tf in leaves might also explain why the yield in transgenic leaves was only slightly lower compared to seeds with 5.6 mg kg^–1^ LFM and 6.7 mg kg^–1^ SDM for SL632 (Table 3), even though the 8- to 30-fold lower protein content in leaves, i.e., 1−2% protein per LFM in leaves compared to 25−30% protein per SDM, would suggest a drastically higher difference, assuming that the activity of the CaMV expression cassette is similar in both tissues. Therefore, transgenic leaves may be a more suitable platform for the production of FGF21-transferrin fusion proteins than seeds. Noteworthy, due to the higher accumulation, transgenic leaves even outcompeted the transient transformation by up to fivefold (Table 3).

Bioavailability and hepatic bioactivity of nTf3338-FGF21-PLUS after oral delivery was evidenced by a first *in vivo* trial using FGF21^–/–^ mice (Figure 4). This is in agreement with previous studies in which partially purified recombinant protein has been used for oral delivery in mice without any obvious negative impact of the plant-related impurities (Choi et al., 2014). Our study also confirmed that Tf can be used to mediate an uptake from the intestine to blood as shown for Ex-4 (Choi et al., 2014), while the PLUS peptide enabled exclusive delivery to the liver as demonstrated for endostatin (Lu et al., 2014; Ma et al., 2014, 2017; Taverner et al., 2020). The observed effects on liver gene expression are in line with the known beneficial hepatic effects of FGF21 such as a reduction of lipid accumulation and improvement of mitochondrial function (Tillman and Rolph, 2020).

## Data availability statement

The original contributions presented in this study are included in the article/Supplementary material, further inquiries can be directed to the corresponding author.

## Ethics statement

The animal study was reviewed and approved by the Ethics Committee of the Ministry for Environment, Health and Consumer Protection of Brandenburg, Germany (approval no. 2347-9-2020).

## Author contributions

H-WH, HN, CB, SK, IB, and JB conceived the experiments. H-WH and CB conducted the experiments and analyzed the data. H-WH and HN wrote the manuscript. All authors contributed to manuscript revision, read, and approved the submitted version.

## Footnotes

1
https://ventria.com/

2
https://www.orfgenetics.com/

3
http://nicota.de/de/index.html

4
https://sunchem.nl/

5
http://raptorx.uchicago.edu/
