# Supplementary material for: Seed- and leaf-based expression of FGF21-transferrin fusion proteins for oral delivery and treatment of non-alcoholic steatohepatitis
Source: Front Plant Sci. 2022 Sep 29;13:998596. doi: 10.3389/fpls.2022.998596 (PMC9557105; doi:10.3389/fpls.2022.998596)
Supplement: Supplementary file 1 [file Data_Sheet_1.docx]

**Supplementary Material**

**Supplementary Tables**

**Table S1. Primers used in this study.**

| **Primer** | **Sequence** |
| --- | --- |
| FGF21_Nt-fw | 5’-TGGACTACCACCAGCATTACCAGAAC-3’ |
| FGF21_Nt-rv | 5’-GTGACCTACCTTGGGATGGACCAAC-3’ |
| Tf_Nt-fw | 5’-CCCCAACTTTGCCAACTCTGTCC-3’ |
| Tf_Nt-rv | 5’-CATTGCGTCAGCCTCCCCATTC-3’ |
| B2m-fw | 5’-CCTTCAGCAAGGACTGGTCT-3’ |
| B2m-rv | 5’-TGTCTCGATCCCAGTAGACG-3’ |
| Srebf1-fw | 5’-GAGGATAGCCAGGTCAAAGC-3’ |
| Srebf1-rv | 5’-AGGATTGCAGGTCAGACACA-3’ |
| Fasn-fw | 5’-TTGATGATTCAGGGAGTGGA-3’ |
| Fasn-rv | 5’-TTACACCTTGCTCCTTGCTG-3’ |
| Ppargc1a-fw | 5’-AGCCGTGACCACTGACAACGAG-3’ |
| Ppargc1a -rv | 5’-GCTGCATGGTTCTGAGTGCTAAG-3’ |
| Ppara-fw | 5’-TGGCAAAGTCTTAGTGCCAGA-3’ |
| Ppara-rv | 5’-TCACTAGGTCACACAGCCTCT-3’ |
| Cpt1a-fw | 5’-CCAAACCCACCAGGCTACA-3’ |
| Cpt1a-rv | 5’-GCACTGCTTAGGGATGTCTCTATG-3’ |

**Table S2. Seed traits SL632 and VG.** Numbers represent mean ± standard deviation. Significance was calculated by one-way ANOVA (Bonferroni) with * p < 0.05, ** p < 0.01 and *** p < 0.001. n=21-25, technical replicates.

| **Parameter** | **Unit** | ***N. tabacum* cultivar** | | |
| --- | --- | --- | --- | --- |
|  |  | **SL632** | **VG** | **Significance** |
| Seed length | µm seed^-1^ | 799.2 ± 48.8 | 672.9 ± 43.6 | *** |
| Seed width | µm seed^-1^ | 535.8 ± 49.4 | 501.4 ± 35.4 | * |
| Thousand SDM | mg cultivar^-1^ | 106.6 ± 4.6 | 82.2 ± 3.8 | *** |

SDM – seed dry mass.

**Table S3. Propagation of *N. tabacum* SL632 and VG.** Numbers represent mean ± standard deviation. n=21, biological replicates.

| **Propagation** | **Unit** | ***N. tabacum* cultivar** | |
| --- | --- | --- | --- |
|  |  | **SL632** | **VG** |
| Average length of passage in tissue culture | day | 21.0 ± 0.0 | 23.1 ± 2.7 |
| Maximum number of passages until 50% of the plants flowered | number | 6th | >19th |

**Table S4. Transgenic T0 events in *N. tabacum* SL632 and VG.** Numbers represent mean ± standard deviation. n=2-3, biological replicates.

| **Stable transformation** | **Unit** | ***N. tabacum* cultivar** | | | |
| --- | --- | --- | --- | --- | --- |
|  |  | **SL632** | | **VG** | |
|  |  | **GFP** | **FGF21-F-Tf** | **GFP** | **FGF21-F-Tf** |
| Shoot/Explant | - | 0.8 ± 0.1 | 1.1 ± 0.3 | 0.3 ± 0.1 | 0.6 ± 0.1 |
| Positive rooted shoots | % | 59% | 62% | 74% | 37% |
| Number of T0 events | number | 23 | 26 | 33 | 26 |

**Table S5. T1 segregation analysis of FGF21-F-Tf in *N. tabacum* SL632 and VG.** Numbers represent mean ± standard deviation. n=3, biological replicates.

| ***N. tabacum* cultivar** | **T1 event** | **Germinating seeds** | **Km-resistant descendants** |
| --- | --- | --- | --- |
| SL632 | NIC | 87 ± 4% | 0% |
|  | 1 | 87 ± 6% | 86 ± 8% |
|  | 27 | 96 ± 4% | 95 ± 5% |
|  | 49 | 92 ± 4% | 93 ± 7% |
| VG | NIC | 85 ± 8% | 0% |
|  | 2 | 97 ± 2% | 92 ± 4% |
|  | 50 | 96 ± 2% | 97 ± 3% |
|  | 51 | 87 ± 6% | 94 ± 4% |

NIC – near-isogenic control plants. Km – kanamycin.

**Table S6. Yield of recombinant proteins in seeds of different crops.** Numbers represent mean ± standard deviation.

| **Plant** | **Recombinant protein** | **Promoter/Terminator** | **Protein accumulation in seed**  **[mg kg^-1^ SDM]** | **Reference** |
| --- | --- | --- | --- | --- |
| Tobacco | FGF21-F-Tf | p35s/t35s | 6.7 ± 0.2 | This study |
|  | Interleukin 6 | p35s/t35s | 303 | (Nausch et al., 2012b) |
|  | scFv antibody | pLeb4/t35s | 230 | (Fiedler and Conrad, 1995) |
|  | Insulin-like growth factor binding protein-3 | pPhas/tPhas | 800 | (Cheung et al., 2009) |
|  | Hemagglutinin | pPhas/tArc | 3,000 | (Ceballo et al., 2017) |
|  | Anti-hepatitis B surface antigen antibody | pPhas/tArc | 6,500 | (Hernandez-Velazquez et al., 2015) |
|  | FLAG multimeric protein | pUSP/t35s | 190 | (Weichert et al., 2016) |
| Rice | Transferrin | pGt1/tNos | 10,000 | (Zhang et al., 2010) |
| Barley | Anti-HIV antibody | pGlo1/tNos | 1,200 | (Hensel et al., 2015) |
| Maize | β‐glucuronidase | pUbi/tPinII | 1,300 | (Kusnadi et al., 1998) |
| Pea | Anti-Eimeria antibody | pUSP/t35s | 1,800 | (Zimmermann et al., 2009) |
| Soybean | Bone morphogenetic proteins 2 | pCon/tCon | 9,300 | (Queiroz et al., 2019) |
| Arabidopsis | murine single chain variable fragment G4 | pPhas/tArc | 10,000-15,000 | (de Jaeger et al., 2002) |

SDM – seed dry mass; p35 – CaMV promoter; pCon – β-conglycinin α-subunit promoter; pGlo1 – *Avena sativa* Globulin 1 promoter; pGt1 – *Oryza sativa* seed storage protein glutenin 1 promoter; pLeb4 *Vicia faba* Legumin B4 promotor of the 11S globulin; pPhas - *Phaseolus vulgaris* promoter of the 7S globulin; pUbi – *Zea mays* upquitin 1 promoter; pUSP – *Vicia faba* unknown seed protein promoter; t35s – CaMV terminator; tArc – *Phaseolus vulgaris* terminator of the arcelin 5-I seed storage protein; tCon – β-conglycinin α-subunit terminator; tNos – *Agrobacterium tumefaciens* nopalin synthase terminator; tPhas - *Phaseolus vulgaris* terminator of the 7S globulin; tPinII - *Solanum tubersosum* potato proteinase inhibitor II terminator.

Ceballo, Y., Tiel, K., Lopez, A., Cabrera, G., Perez, M., Ramos, O., Rosabal, Y., Montero, C., Menassa, R., Depicker, A. and Hernandez, A. (2017). High accumulation in tobacco seeds of hemagglutinin antigen from avian (H5N1) influenza. *Transgenic Res.* **26**, 775-789.

Cheung, S. C., Sun, S. S., Chan, J. C. and Tong, P. C. (2009). Expression and subcellular targeting of human insulin-like growth factor binding protein-3 in transgenic tobacco plants. *Transgenic Res.* **18**, 943-951.

De Jaeger, G., Scheffer, S., Jacobs, A., Zambre, M., Zobell, O., Goossens, A., Depicker, A. and Angenon, G. (2002). Boosting heterologous protein production in transgenic dicotyledonous seeds using *Phaseolus vulgaris* regulatory sequences. *Nat Biotechnol.* **20**, 1265-1268.

Fiedler, U. and Conrad, U. (1995). High-level production and long-term storage of engineered antibodies in transgenic tobacco seeds. *Biotechnology.* **13**, 1090-1093

Hensel, G., Floss, D. M., Arcalis, E., Sack, M., Melnik, S., Altmann, F., Rutten, T., Kumlehn, J., Stoger, E. and Conrad, U. (2015). Transgenic Production of an Anti HIV Antibody in the Barley Endosperm. *PLoS One.* **10**, e0140476.

Hernandez-Velazquez, A., Lopez-Quesada, A., Ceballo-Camara, Y., Cabrera-Herrera, G., Tiel-Gonzalez, K., Mirabal-Ortega, L., Perez-Martinez, M., Perez-Castillo, R., Rosabal-Ayan, Y., Ramos-Gonzalez, O., Enriquez-Obregon, G., Depicker, A. and Pujol-Ferrer, M. (2015). Tobacco seeds as efficient production platform for a biologically active anti-HBsAg monoclonal antibody. *Transgenic Res.* **24**, 897-909.

Kusnadi, A. R., Hood, E. E., Witcher, D. R., Howard, J. A. and Nikolov, Z. L. (1998). Production and purification of two recombinant proteins from transgenic corn. *Biotechnol Prog.* **14**, 149-155.

Nausch, H., Mikschofsky, H., Koslowski, R., Meyer, U., Broer, I. and Huckauf, J. (2012b). High-level transient expression of ER-targeted human interleukin 6 in *Nicotiana benthamiana*. *PLoS One.* **7**, e48938.

Queiroz, L. N., Maldaner, F. R., Mendes, É. A., Sousa, A. R., D'Allastta, R. C., Mendonça, G., Mendonça, D. B. S. and Aragão, F. J. L. (2019). Evaluation of lettuce chloroplast and soybean cotyledon as platforms for production of functional bone morphogenetic protein 2. *Transgenic Res.* **28**, 213-224.

Weichert, N., Hauptmann, V., Helmold, C. and Conrad, U. (2016). Seed-Specific Expression of Spider Silk Protein Multimers Causes Long-Term Stability. *Front Plant Sci*. **7**, e6.

Zhang, D., Nandi, S., Bryan, P., Pettit, S., Nguyen, D., Santos, M. A. and Huang, N. (2010). Expression, purification, and characterization of recombinant human transferrin from rice (*Oryza sativa L.*). *Protein Expr Purif*. **74**, 69-79.

Zimmermann, J., Saalbach, I., Jahn, D., Giersberg, M., Haehnel, S., Wedel, J., Macek, J., Zoufal, K., Glünder, G., Falkenburg, D. and Kipriyanov, S. M. (2009). Antibody expressing pea seeds as fodder for prevention of gastrointestinal parasitic infections in chickens. *BMC Biotechnol.* **9**, 79-100.

**Table S7. Linkers described for FGF21-transferrin fusion proteins.**

| **Linker** | **Fusion protein** | **Heterologous host** | **Compartment** | **Reference** |
| --- | --- | --- | --- | --- |
| DDDDK* | GFP-FGF21 | *Nicotiana benthamiana* | Cytosol | (Fu et al., 2011) |
| LE** | ProINS-Tf | HEK293 | Extracellular space | (Wang et al., 2011; Wang et al., 2014; Chen et al., 2018; Liu et al., 2020) |
|  |  | *Oryza sativa* seeds | Apoplasm | (Chen et al., 2018) |
| (PEAPTD)_2_ | GLP1-Tf | *Saccharomyces cerevisiae* | Extracellular space | (Kim et al., 2010) |
|  | Ex4-Tf | *Saccharomyces cerevisiae* | Extracellular space | (Kim et al., 2010) |
| (LEA(EAAAK)_4_ALEA  (EAAAK)_4_ALE) | G-CSF-Tf | HEK293 | Extracellular space | (Amet et al., 2009; Chen et al., 2012) |
|  | FIX-Tf | HEK293 | Extracellular space | (Amet et al., 2009; Chen et al., 2012) |
| (GGGGS)_3_ | G-CSF-Tf | HEK293 | Extracellular space | (Amet et al., 2009; Chen et al., 2012) |
|  | FIX-Tf | HEK293 | Extracellular space | (Amet et al., 2009; Chen et al., 2012) |
|  | Ex4-Tf | *Nicotiana benthamiana* | ER | (Choi et al., 2014) |

*enterokinase cleavage site. **XhoI – restriction site.

Amet, N., Lee, H.F. and Shen, W.C. (2009). Insertion of the designed helical linker led to increased expression of Tf-based fusion proteins. *Pharm* *Res.* **26**, 523-528.

Chen, X., Zaro, J. L. and Shen, W. C. (2012). Fusion Protein Linkers: Property, Design and Functionality. *Adv Drug Deliv Rev.* **65**, 1357-1369.

Chen, Y. S., Zaro, J. L., Zhang, D., Huang, N., Simon, A. and Shen, W. C. (2018). Characterization and Oral Delivery of Proinsulin-Transferrin Fusion Protein Expressed Using ExpressTec. *Int J Mol Sci.* **19**, 378-390.

Choi, J., Diao, H., Feng, Z.-C., Lau, A., Wang, R., Jevnikar, A. M. and Ma, S. (2014) A fusion protein derived from plants holds promising potential as a new oral therapy for type 2 diabetes. *Plant Biotechnol J.* **12**, 425-435.

Fu, H., Pang, S., Xue, P., Yang, J., Liu, X., Wang, Y., Li, T., Li, H. and Li, X. (2011). High levels of expression of fibroblast growth factor 21 in transgenic tobacco (*Nicotiana benthamiana*). *Appl Biochem Biotechnol.* **165**, 465-475.

Kim, B. J., Zhou, J., Martin, B., Carlson, O. D., Maudsley, S., Greig, N. H., Mattson, M. P., Ladenheim, E. E., Wustner, J., Turner, A., Sadeghi, H. and Egan, J. M. (2010). Transferrin fusion technology: a novel approach to prolonging biological half-life of insulinotropic peptides. *J Pharmacol Exp Ther.* **334**, 682-692.

Liu, Y., Wang, H. Y., Shao, J., Zaro, J. L. and Shen, W. C. (2020). Enhanced insulin receptor interaction by a bifunctional insulin-transferrin fusion protein: an approach to overcome insulin resistance. *Sci Rep.* **10**, e7724.

Wang, Y., Chen, Y. S., Zaro, J. L. and Shen, W. C. (2011). Receptor-Mediated Activation of a Proinsulin-Transferrin Fusion Protein in Hepatoma Cells. *J Control Release.* **155**, 386-392.

Wang, Y., Shao, J., Zaro, J. L. and Shen, W. C. (2014). Proinsulin-Transferrin Fusion Protein as a Novel Long-Acting Insulin Analog for the Inhibition of Hepatic Glucose Production. *Diabetes.* **63**, 1779-1788.

**Supplementary Figures**

**>p9U-FGF21-F-Tf**

MATQRRANPSSLHLITVFSLLVAVVSGHPIPDSSPLLQFGGQVRQRYLYTDDAQQTEAHLEIREDGTVGGAADQSPESLLQLKALKPGVIQILGVKTSRFLCQRPDGALYGSLHFDPEACSFRELLLEDGYNVYQSEAHGLPLHLPGNKSPHRDPAPRGPARFLPLPGLPPALPEPPGILAPQPPDVGSSDPLAMVGPSQGRSPSYASRRKRSVGGGGSGGGGSGGGGSVPDKTVRWCAVSEHEATKCQSFRDHMKSVIPSDGPSVACVKKASYLDCIRAIAANEADAVTLDAGLVYDAYLAPNNLKPVVAEFYGSKEDPQTFYYAVAVVKKDSGFQMNQLRGKKSCHTGLGRSAGWNIPIGLLYCDLPEPRKPLEKAVANFFSGSCAPCADGTDFPQLCQLCPGCGCSTLNQYFGYSGAFKCLKDGAGDVAFVKHSTIFENLANKADRDQYELLCLDNTRKPVDEYKDCHLAQVPSHTVVARSMGGKEDLIWELLNQAQEHFGKDKSKEFQLFSSPHGKDLLFKDSAHGFLKVPPRMDAKMYLGYEYVTAIRNLREGTCPEAPTDECKPVKWCALSHHERLKCDEWSVNSVGKIECVSAETTEDCIAKIMNGEADAMSLDGGFVYIAGKCGLVPVLAENYNKSDNCEDTPEAGYFAIAVVKKSASDLTWDNLKGKKSCHTAVGRTAGWNIPMGLLYNKINHCRFDEFFSEGCAPGSKKDSSLCKLCMGSGLNLCEPNNKEGYYGYTGAFRCLVEKGDVAFVKHQTVPQNTGGKNPDPWAKNLNEKDYELLCLDGTRKPVEEYANCHLARAPNHAVVTRKDKEACVHKILRQQQHLFGSNVTDCSGNFCLFRSETKDLLFRDDTVCLAKLHDRNTYEKYLGEEYVKAVGNLRKCSTSSLLEACTFRRPLEHHHHHHSRAWRHPQFGGHHHHHHSEKDEL*

**>pICH29912-FGF21-F-IntN**

MATQRRANPSSLHLITVFSLLVAVVSGHPIPDSSPLLQFGGQVRQRYLYTDDAQQTEAHLEIREDGTVGGAADQSPESLLQLKALKPGVIQILGVKTSRFLCQRPDGALYGSLHFDPEACSFRELLLEDGYNVYQSEAHGLPLHLPGNKSPHRDPAPRGPARFLPLPGLPPALPEPPGILAPQPPDVGSSDPLAMVGPSQGRSPSYASRRKRSVRESGCISGDSLISLASTGKRVSIKDLLDEKDFEIWAINEQTMKLESAKVSRVFCTGKKLVYILKTRLGRTIKATANHRFLTIDGWKRLDELSLKEHIALPRKLESSSLQLGLEHHHHHHSRAWRHPQFGGHHHHHHSEKDEL*

**>pICH31120-IntC-Tf**

MATQRRANPSSLHLITVFSLLVAVVSGSPEIEKLSQSDIYWDSIVSITETGVEEVFDLTVPGPHNFVANDIIVHNSIEQDGGGGSGGGGSGGGGSVPDKTVRWCAVSEHEATKCQSFRDHMKSVIPSDGPSVACVKKASYLDCIRAIAANEADAVTLDAGLVYDAYLAPNNLKPVVAEFYGSKEDPQTFYYAVAVVKKDSGFQMNQLRGKKSCHTGLGRSAGWNIPIGLLYCDLPEPRKPLEKAVANFFSGSCAPCADGTDFPQLCQLCPGCGCSTLNQYFGYSGAFKCLKDGAGDVAFVKHSTIFENLANKADRDQYELLCLDNTRKPVDEYKDCHLAQVPSHTVVARSMGGKEDLIWELLNQAQEHFGKDKSKEFQLFSSPHGKDLLFKDSAHGFLKVPPRMDAKMYLGYEYVTAIRNLREGTCPEAPTDECKPVKWCALSHHERLKCDEWSVNSVGKIECVSAETTEDCIAKIMNGEADAMSLDGGFVYIAGKCGLVPVLAENYNKSDNCEDTPEAGYFAIAVVKKSASDLTWDNLKGKKSCHTAVGRTAGWNIPMGLLYNKINHCRFDEFFSEGCAPGSKKDSSLCKLCMGSGLNLCEPNNKEGYYGYTGAFRCLVEKGDVAFVKHQTVPQNTGGKNPDPWAKNLNEKDYELLCLDGTRKPVEEYANCHLARAPNHAVVTRKDKEACVHKILRQQQHLFGSNVTDCSGNFCLFRSETKDLLFRDDTVCLAKLHDRNTYEKYLGEEYVKAVGNLRKCSTSSLLEACTFRRPLEHHHHHHSRAWRHPQFGGHHHHHHSEKDEL*

**>pTRAc-FGF21-F-Tf**

MATQRRANPSSLHLITVFSLLVAVVSGHPIPDSSPLLQFGGQVRQRYLYTDDAQQTEAHLEIREDGTVGGAADQSPESLLQLKALKPGVIQILGVKTSRFLCQRPDGALYGSLHFDPEACSFRELLLEDGYNVYQSEAHGLPLHLPGNKSPHRDPAPRGPARFLPLPGLPPALPEPPGILAPQPPDVGSSDPLAMVGPSQGRSPSYASRRKRSVGGGGSGGGGSGGGGSVPDKTVRWCAVSEHEATKCQSFRDHMKSVIPSDGPSVACVKKASYLDCIRAIAANEADAVTLDAGLVYDAYLAPNNLKPVVAEFYGSKEDPQTFYYAVAVVKKDSGFQMNQLRGKKSCHTGLGRSAGWNIPIGLLYCDLPEPRKPLEKAVANFFSGSCAPCADGTDFPQLCQLCPGCGCSTLNQYFGYSGAFKCLKDGAGDVAFVKHSTIFENLANKADRDQYELLCLDNTRKPVDEYKDCHLAQVPSHTVVARSMGGKEDLIWELLNQAQEHFGKDKSKEFQLFSSPHGKDLLFKDSAHGFLKVPPRMDAKMYLGYEYVTAIRNLREGTCPEAPTDECKPVKWCALSHHERLKCDEWSVNSVGKIECVSAETTEDCIAKIMNGEADAMSLDGGFVYIAGKCGLVPVLAENYNKSDNCEDTPEAGYFAIAVVKKSASDLTWDNLKGKKSCHTAVGRTAGWNIPMGLLYNKINHCRFDEFFSEGCAPGSKKDSSLCKLCMGSGLNLCEPNNKEGYYGYTGAFRCLVEKGDVAFVKHQTVPQNTGGKNPDPWAKNLNEKDYELLCLDGTRKPVEEYANCHLARAPNHAVVTRKDKEACVHKILRQQQHLFGSNVTDCSGNFCLFRSETKDLLFRDDTVCLAKLHDRNTYEKYLGEEYVKAVGNLRKCSTSSLLEACTFRRPLEHHHHHHSRAWRHPQFGGHHHHHHSEKDEL*

**>pTRAc-FGF21-Tf-PLUS**

MATQRRANPSSLHLITVFSLLVAVVSGHPIPDSSPLLQFGGQVRQRYLYTDDAQQTEAHLEIREDGTVGGAADQSPESLLQLKALKPGVIQILGVKTSRFLCQRPDGALYGSLHFDPEACSFRELLLEDGYNVYQSEAHGLPLHLPGNKSPHRDPAPRGPARFLPLPGLPPALPEPPGILAPQPPDVGSSDPLAMVGPSQGRSPSYASGGGGSGGGGSGGGGSVPDKTVRWCAVSEHEATKCQSFRDHMKSVIPSDGPSVACVKKASYLDCIRAIAANEADAVTLDAGLVYDAYLAPNNLKPVVAEFYGSKEDPQTFYYAVAVVKKDSGFQMNQLRGKKSCHTGLGRSAGWNIPIGLLYCDLPEPRKPLEKAVANFFSGSCAPCADGTDFPQLCQLCPGCGCSTLNQYFGYSGAFKCLKDGAGDVAFVKHSTIFENLANKADRDQYELLCLDNTRKPVDEYKDCHLAQVPSHTVVARSMGGKEDLIWELLNQAQEHFGKDKSKEFQLFSSPHGKDLLFKDSAHGFLKVPPRMDAKMYLGYEYVTAIRNLREGTCPEAPTDECKPVKWCALSHHERLKCDEWSVNSVGKIECVSAETTEDCIAKIMNGEADAMSLDGGFVYIAGKCGLVPVLAENYNKSDNCEDTPEAGYFAIAVVKKSASDLTWDNLKGKKSCHTAVGRTAGWNIPMGLLYNKINHCRFDEFFSEGCAPGSKKDSSLCKLCMGSGLNLCEPNNKEGYYGYTGAFRCLVEKGDVAFVKHQTVPQNTGGKNPDPWAKNLNEKDYELLCLDGTRKPVEEYANCHLARAPNHAVVTRKDKEACVHKILRQQQHLFGSNVTDCSGNFCLFRSETKDLLFRDDTVCLAKLHDRNTYEKYLGEEYVKAVGNLRKCSTSSLLEACTFRRPDNEKLRKPKHKKLKQPADGLEHHHHHHSRAWRHPQFGGHHHHHHSEKDEL*

**>pTRAc-FGF21-nTf338-PLUS**

MATQRRANPSSLHLITVFSLLVAVVSGHPIPDSSPLLQFGGQVRQRYLYTDDAQQTEAHLEIREDGTVGGAADQSPESLLQLKALKPGVIQILGVKTSRFLCQRPDGALYGSLHFDPEACSFRELLLEDGYNVYQSEAHGLPLHLPGNKSPHRDPAPRGPARFLPLPGLPPALPEPPGILAPQPPDVGSSDPLAMVGPSQGRSPSYASGGGGSGGGGSGGGGSVPDKTVRWCAVSEHEATKCQSFRDHMKSVIPSDGPSVACVKKASYLDCIRAIAANEADAVTLDAGLVYDAYLAPNNLKPVVAEFYGSKEDPQTFYYAVAVVKKDSGFQMNQLRGKKSCHTGLGRSAGWNIPIGLLYCDLPEPRKPLEKAVANFFSGSCAPCADGTDFPQLCQLCPGCGCSTLNQYFGYSGAFKCLKDGAGDVAFVKHSTIFENLANKADRDQYELLCLDNTRKPVDEYKDCHLAQVPSHTVVARSMGGKEDLIWELLNQAQEHFGKDKSKEFQLFSSPHGKDLLFKDSAHGFLKVPPRMDAKMYDNEKLRKPKHKKLKQPADGLEHHHHHHSRAWRHPQFGGHHHHHHSEKDEL*

**>pTRAc-nTf338-FGF21-PLUS**

MATQRRANPSSLHLITVFSLLVAVVSGVPDKTVRWCAVSEHEATKCQSFRDHMKSVIPSDGPSVACVKKASYLDCIRAIAANEADAVTLDAGLVYDAYLAPNNLKPVVAEFYGSKEDPQTFYYAVAVVKKDSGFQMNQLRGKKSCHTGLGRSAGWNIPIGLLYCDLPEPRKPLEKAVANFFSGSCAPCADGTDFPQLCQLCPGCGCSTLNQYFGYSGAFKCLKDGAGDVAFVKHSTIFENLANKADRDQYELLCLDNTRKPVDEYKDCHLAQVPSHTVVARSMGGKEDLIWELLNQAQEHFGKDKSKEFQLFSSPHGKDLLFKDSAHGFLKVPPRMDAKMYGGGGSGGGGSGGGGSHPIPDSSPLLQFGGQVRQRYLYTDDAQQTEAHLEIREDGTVGGAADQSPESLLQLKALKPGVIQILGVKTSRFLCQRPDGALYGSLHFDPEACSFRELLLEDGYNVYQSEAHGLPLHLPGNKSPHRDPAPRGPARFLPLPGLPPALPEPPGILAPQPPDVGSSDPLAMVGPSQGRSPSYASDNEKLRKPKHKKLKQPADGLEHHHHHHSRAWRHPQFGGHHHHHHSEKDEL*

**Figure S1. Amino acids sequences of FGF21-transferrin fusion proteins used for the stable and transient expression.** *Light blue*: ER-targeting signal peptide from Calreticulin of *Nicotiana plumbaginifolia* (Acc. Z71395); *Orange*: FGF21 mature protein with Ser167Ala mutation (underlined) to eliminate the O-glycosylation site; *Green*: Furin cleavage site to separate FGF21 from transferrin in the enterocytes to release FGF21 into the blood serum; *Light red:* GS-Linker to separate FGF21 and Tf; *Black*: Transferrin mature protein (n-terminal domain nTf338 double underlined); *Dark purple*: double His6-Tag; *Dark blue*: ER retention signal; *Dark Red*: Extensin from DnaB for transplicing; *Light purple*: PLUS liver targeting peptide of the CSP protein.

**
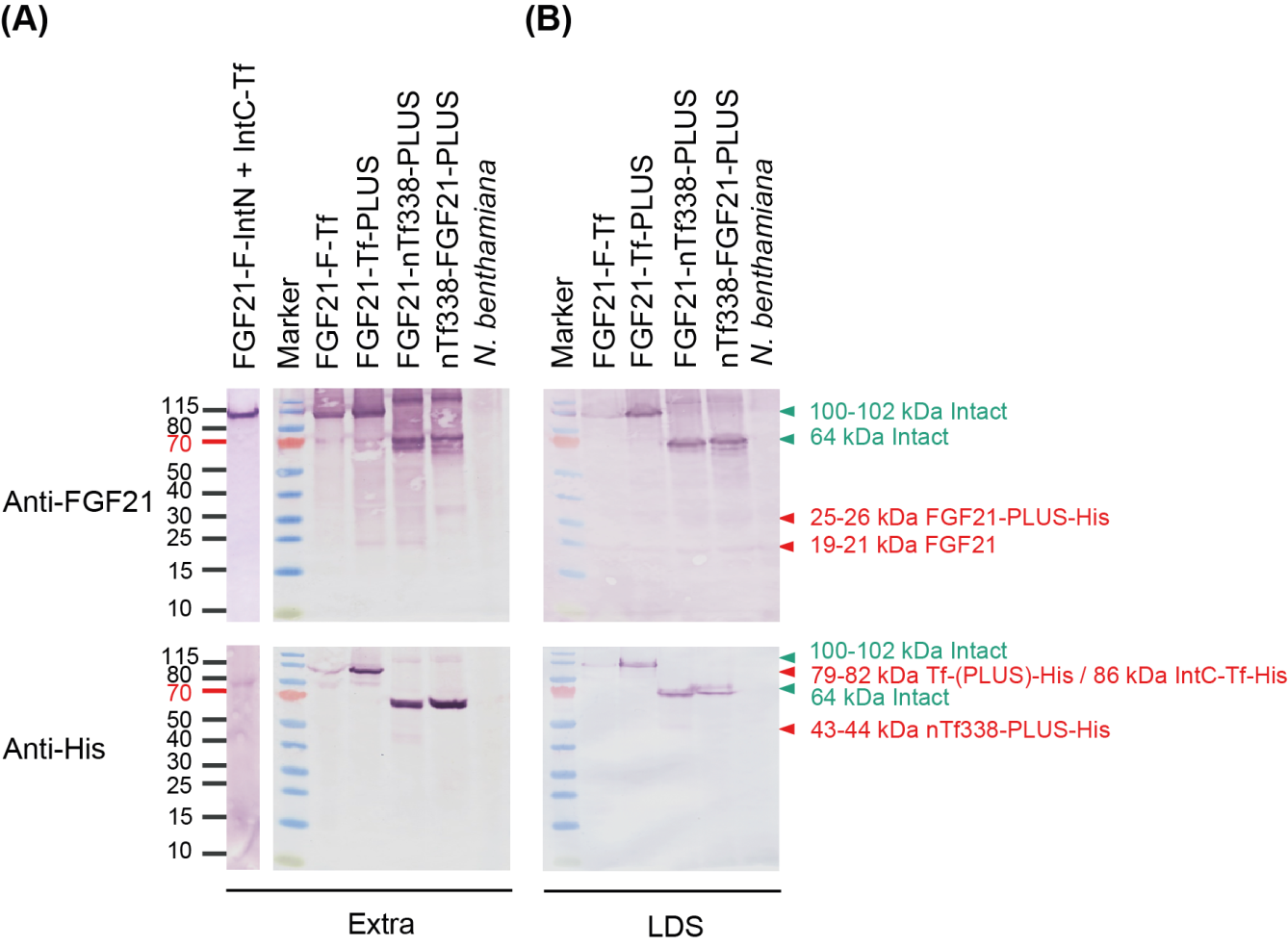
**

**Figure S2. Western blot analysis of modified FGF21-transferrin fusion proteins produced in *N. benthamiana* using magnICON and pTRAc systems.** MagnICON transient expression: co-expression of FGF21-F-IntN plus IntC-Tf. and pTRAc transient system: single expression of FGF21-F-Tf, FGF21-Tf-PLUS, FGF21-nTf338-PLUS and nTf338-FGF21-PLUS. Each 100 mg leaf materials were extracted in (A) extraction buffer (Extra) or (B) LDS buffer (LDS). Leaf crude extracts under extraction and LDS buffer containing 100 μg of total soluble protein and 20 μL of total volume were subjected to NuPAGE 4–12% Bis-Tris protein gels, respectively. The electroblotted proteins were probe with primary anti-FGF21/-His6 rabbit polyclonal antibody (1:5,000) and then secondary goat anti-Rabbit IgG alkaline phosphate (AP)-conjugated antibody (1:5,000). *Green arrows*: intact fusion protein, *red arrows*: degraded fusion protein.


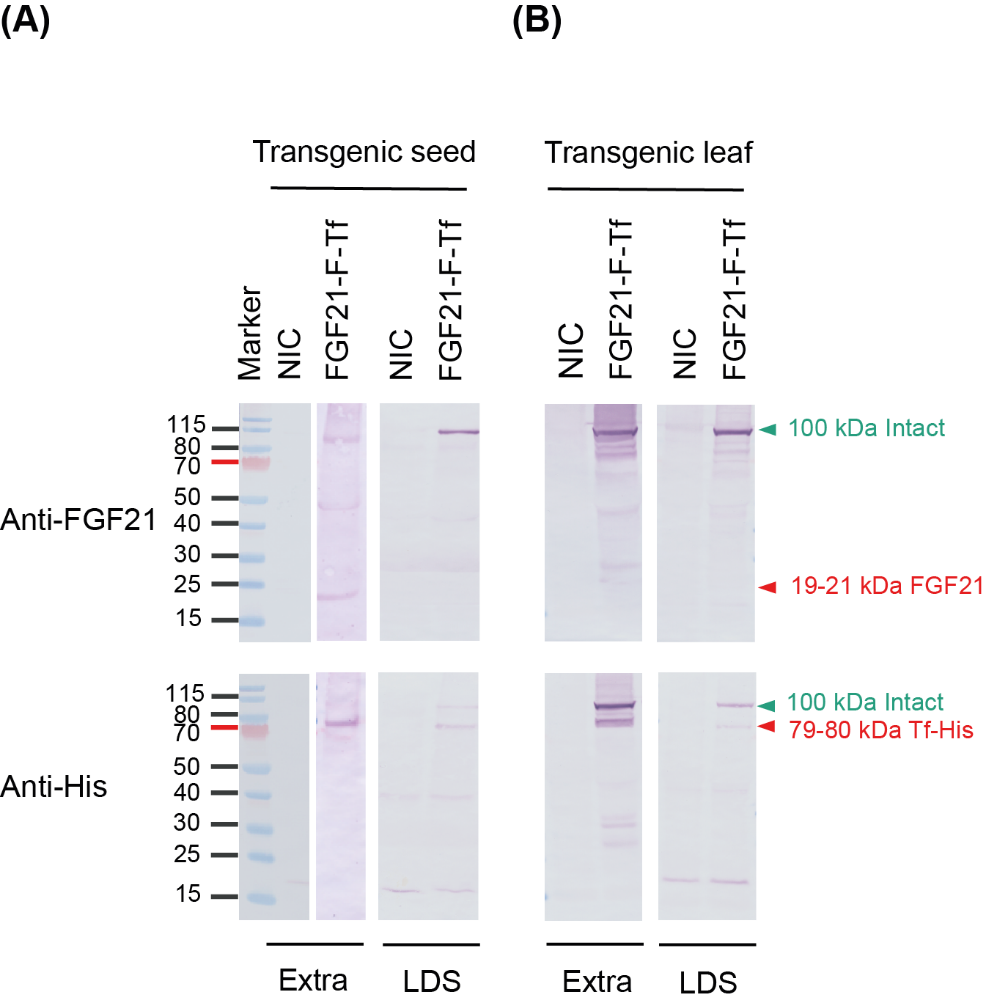


**Figure S3. Western blot analysis of FGF21-transferrin fusion proteins in *N. tabacum* SL632.** Transgenic seed (A) and transgenic leaf (B). Each 100 mg plant material was extracted in extraction buffer (Extra) or LDS buffer (LDS). Seed and leaf crude extracts under extraction and LDS buffer containing 100 μg of total soluble protein and 20 μL of total volume were subjected to NuPAGE 4–12% Bis-Tris protein gels, respectively. The electroblotted proteins were probe with primary anti-FGF21/-His6 rabbit polyclonal antibody (1:5,000) and then secondary goat anti-Rabbit IgG alkaline phosphate (AP)-conjugated antibody (1:5,000). *Green arrows*: intact fusion protein, *red arrows*: degraded fusion protein.


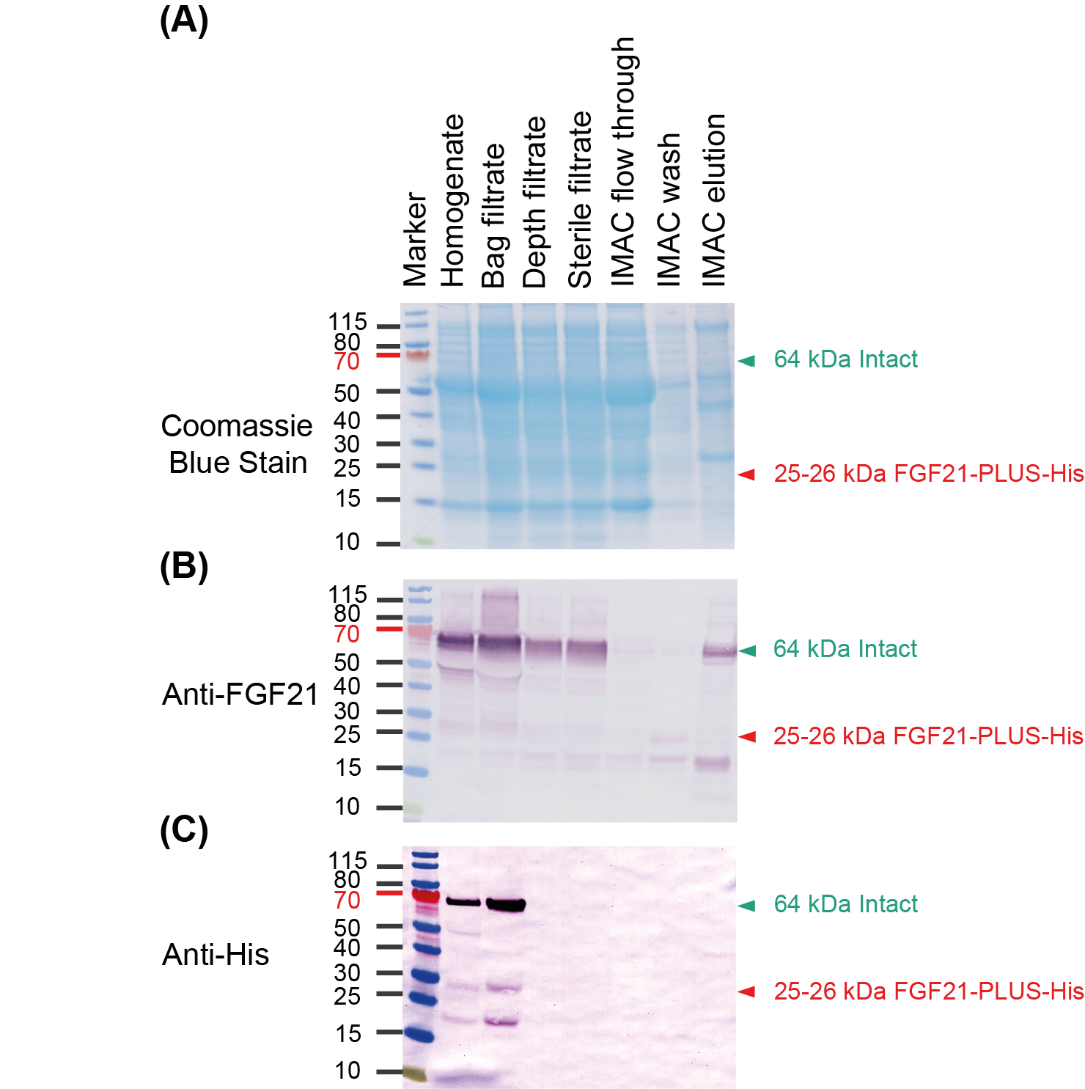


**Figure S4.** **Coomassie-stained NuPAGE LDS gel and Western blot analysis of purification process fractions for nTF338-FGF21-PLUS extracted from non-senescent, transiently transformed *N. benthamiana* leaves, using depth filtration for clarification.** Each 800 g leaf material was homogenized in extraction buffer and purified by Immobilized Metal Ion Affinity Chromatography (IMAC). Leaf samples containing 100 μg of total soluble protein (TSP) were subjected to NuPAGE 4–12% Bis-Tris protein gels. (A) Coomassie stained gel, (B) FGF21-Western blot and (C) His-Western blot. The electroblotted proteins were probed with primary anti-FGF21/-His6 rabbit polyclonal antibody (1:5,000) and then secondary goat anti-Rabbit IgG alkaline phosphate (AP)-conjugated antibody (1:5,000). *Green arrows*: intact fusion protein, *red arrows*: degraded fusion protein.


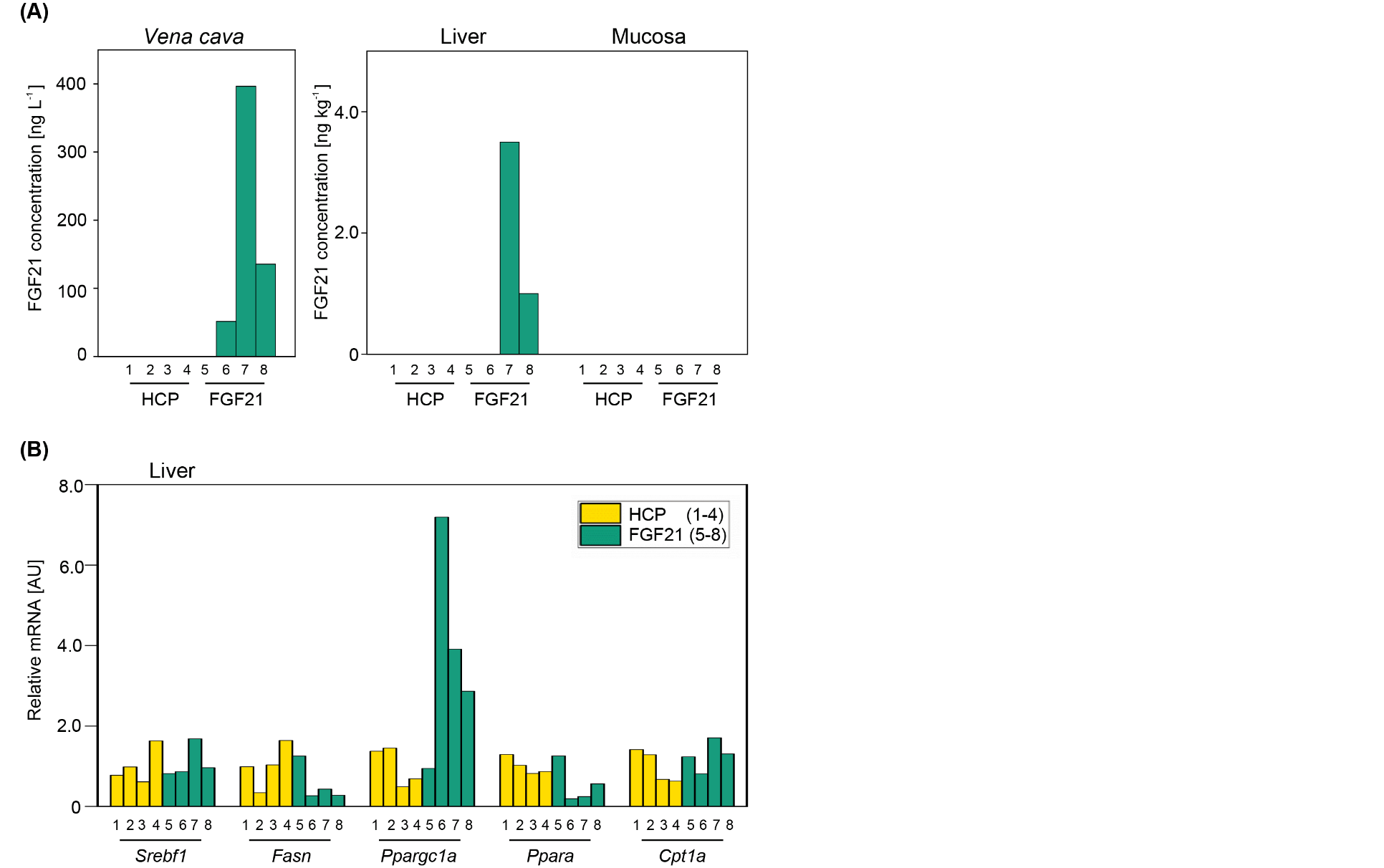


**Figure S5.** **The bioavailability and bioactivity of the purified fusion protein nTf338-FGF21-PLUS of individual mice in an *in vivo* animal bolus feeding trial after 4 h.** FGF21^-/-^ knockout mice were starved for 16 h and gavaged with a 0.5 mL bolus of partially purified nTf338-FGF21-PLUS dissolved in water to a concentration of 50 µg L^-1^ FGF21 and 44 g L^-1^ HCP (FGF21 group) or HCP only (HCP group). After 4 h, mice were killed. (A) FGF21 concentration in the *Vena cava*, liver and mucosa of via FGF21-ELISA. (B) mRNA expression levels of the gene *Srebf1*, *Fasn*, *Ppargc1a*, *Ppara* and *Cpt1a* were determined in liver tissue via qPCR (primer are listed in Table S7). Mice 1-4: feeded with HCP (IMAC-purified plant host cell proteins from *N. benthamiana* leaves), mice 5-8: gavaged with FGF21 (IMAC-purified nTf338-FGF21-PLUS from *N. benthamiana* leaves).
